# Structural and functional multi-platform MRI series of a single human volunteer over more than fifteen years

**DOI:** 10.1038/s41597-019-0262-8

**Published:** 2019-10-31

**Authors:** Simon Duchesne, Louis Dieumegarde, Isabelle Chouinard, Farnaz Farokhian, Amanpreet Badhwar, Pierre Bellec, Pascal Tétreault, Maxime Descoteaux, Arnaud Boré, Jean-Christophe Houde, Christian Beaulieu, Olivier Potvin

**Affiliations:** 1 Department of Radiology, Université Laval, Québec, Canada; 2 CERVO Brain Research Centre, Institut universitaire de santé mentale de Québec, Québec, Canada; 3 Centre de recherche de l’Institut universitaire en gériatrie de Montréal, Québec, Canada; 4 Department of Biomedical Engineering, Faculty of Medicine and Dentistry, University of Alberta, Edmonton, Canada; 5 Sherbrooke Connectivity Imaging Lab (SCIL), Université de Sherbrooke, Sherbrooke, Canada

**Keywords:** Neuroscience, Magnetic resonance imaging, Brain imaging

## Abstract

We present MRI data from a single human volunteer consisting in over 599 multi-contrast MR images (T1-weighted, T2-weighted, proton density, fluid-attenuated inversion recovery, T2* gradient-echo, diffusion, susceptibility-weighted, arterial-spin labelled, and resting state BOLD functional connectivity imaging) acquired in over 73 sessions on 36 different scanners (13 models, three manufacturers) over the course of 15+ years (*cf*. Data records). Data included planned data collection acquired within the Consortium pour l’identification précoce de la maladie Alzheimer - Québec (CIMA-Q) and Canadian Consortium on Neurodegeneration in Aging (CCNA) studies, as well as opportunistic data collection from various protocols. These multiple within- and between-centre scans over a substantial time course of a single, cognitively healthy volunteer can be useful to answer a number of methodological questions of interest to the community.

## Abstract (summary)

**Table:** 

Measurement(s)	brain
Technology Type(s)	magnetic resonance imaging
Sample Characteristic - Organism	Homo sapiens

Machine-accessible metadata file describing the reported data: 10.6084/m9.figshare.9925037

## Background & Summary

Reproducibility is a cornerstone scientific principle, especially so when one is faced with measuring instruments that do not produce absolute quantifiable data such as magnetic resonance imaging (**MRI**). The resulting signal varies systemically based on either the type and parameters of the acquisition being chosen, as well as randomly due to the instrument itself. These effects produce changes in signal (i.e. images) that may reach statistical significance when compared to the effect under study. Calibration is therefore needed.

Geometric objects (“phantoms”) have been proposed to track and correct some of these effects, and are presently in routine use. However, there is no geometric phantom however is able to approximate with sufficient fidelity the intricacies of living organs, especially in the case of the human brain, nor mimic its physiological characteristics. To this end, a number of studies have proposed and are using human volunteer(s) as calibration devices, postulating that the time course for changes in any one of the volunteers’ brain will be of smaller magnitude than the changes being measured; in effect, that these brains can be considered nearly identical to themselves across time (the length of the study) and space (scanners and sites).

There have been many instances of human volunteer brain data made accessible for public use. One of the earliest known is referred to as Colin27^1,2^ and consisted in 27 different T1-, T2- and proton-density weighted (**T1w**, **T2w**, **PD**) MRI acquisitions of a single individual, acquired on a single scanner within weeks (http://www.bic.mni.mcgill.ca/ServicesAtlases/Colin27). Table 1 details other works that have been done since, by Brown *et al*.^3^, Friedman *et al*.^4^, Han *et al*.^5^, Suckling *et al*.^6^, and in these pages, efforts coordinated via the Consortium for Data reliability and reproducibility (10.15387/fcp_indi.retro.simon) by Maclaren *et al*.^7^, and Wakeman *et al*.^8^. While extremely useful, one notices a need for a dataset of individual(s) with longitudinal acquisitions of a multimodal protocol using a variety of scanners models and vendors. Such a dataset would serve the needs of multiple imaging researchers to test the reproducibility and robustness of their methods.

**Table 1:** Non-exhaustive list of publicly available human phantom data.

Author	Subjects	Sites	Time range	Repeat	Acquisitions	Harmonized protocol
Brown^3^	18	4	NR	No	BOLD fMRI	No
Friedman^4^	5	10	2 days	Yes	BOLD fMRI	No
Han^5^	15	3	2 weeks	Yes	T1w	No
Holmes^1^	1	1	1 month	27	T1w, T2w, PD	ICBM
Maclaren^7^	3	1	31 days	40	T1w	ADNI
Suckling^6^	12	5	7.8 months (1.9)	Yes	T1w, BOLD fMRI, rsfMRI	No
Wakeman^8^	19	1	3 months	Yes	T1w, FLASH, DI, EEG, MEG	No

NR: not reported.

Our goal was to fill this gap by contributing data from a single human volunteer originating from two settings^9^. In the first, a planned data collection is reported as the single participant acted as a human volunteer for a number of multi-centric studies that began coordinated data acquisition using a trans-national harmonized protocol. The second is an opportunistic data collection with various protocols, as this same volunteer participated in a number of unrelated academic studies, protocol tests, and manufacturer visits (2001-present). Hence this second collection was acquired in a disharmonized fashion. *In fine*, the dataset consists of over 599 multi-contrast MR images (T1w, T2w, PD, fluid-attenuated inversion recovery (**FLAIR**), gradient-echo (T2*), diffusion-weighted (**DWI**), susceptibility-weighted (**SWI**), arterial-spin labeling (**ASL**), time-of-flight (**ToF**) and resting state BOLD functional connectivity imaging (**rsfMRI**). This dataset was acquired through 73 sessions on 36 different scanners (13 models, three manufacturers) over the course of the last 17 years (*cf*. Data records). Given these multiple within- and between-centre scans over a substantial time course, we propose that this data can be useful in answering a number of methodological questions of interest to the community (*cf*. Usage Notes).

## Methods

### Participant and ethical agreement

A Single Individual volunteering for Multiple Observations across Networks (**SIMON**) took part in these studies. SIMON is an ambidextrous male, aged between 28 to 46 years old across the timeline of the dataset, and remains free from known cognitive impairment, neurological disease or psychiatric disorders. Ethical agreements for the purposive acquisitions were obtained from the coordinating ethics committee of either the Institut universitaire de gériatrie de Montréal (*Consortium pour l’identification précoce de la maladie d’Alzheimer – Québec* study **(CIMA-Q)**); the Lady Davis Institute (*Canadian consortium for neurodegeneration and aging* study **(CCNA)**); or of the *Ontario Brain Institute’s Ontario Neurodegenerative Disease Research Initiative* (**ONDRI**)^10^. Ethical agreements for the convenience acquisitions were obtained by each individual investigator at their respective institutions. The participant gave his written informed consent before enrollment in each study and has consented to release his data without restriction.

Part of the data used in this article were obtained from the Consortium pour l’identification précoce de la maladie Alzheimer - Québec (CIMA-Q), founded in 2013 with a $2,500,000 grant from the *Fonds d’Innovation Pfizer - Fond de Recherche Québec – Santé sur la maladie d’Alzheimer et les maladies apparentées*. The main objective is to build a cohort of participants characterized in terms of cognition, neuroimaging and clinical outcomes in order to acquire biological samples allowing (1) to establish early diagnoses of Alzheimer’s disease, (2) to provide a well characterized cohort and (3) to identify new therapeutic targets. The principal investigator and director of CIMA-Q is Dr Sylvie Belleville of the Centre de recherche de l’Institut universitaire de gériatrie de Montréal, CIUSSS Centre-sud-de-l’Île-de-Montréal. CIMA-Q represent a common effort of several researchers from Québec affiliated with Université Laval, McGill University, Université de Montréal, and Université de Sherbrooke. CIMA-Q recruited 350 cognitively healthy participants, with subjective cognitive impairment, mild cognitive impairment, or Alzheimer’s disease, between 2013–2016.

### Purposive acquisitions - MRIs from the canadian dementia imaging protocol

**Fig. 1:**
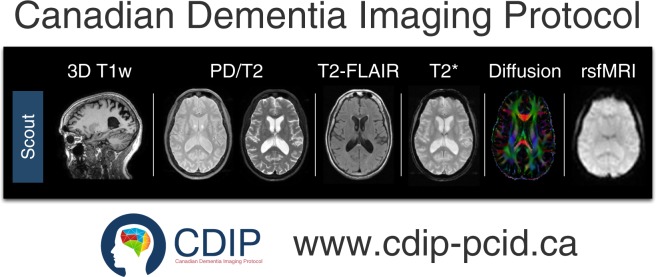
Overview of the Canadian Dementia Imaging Protocol (CDIP).

In Canada, a collection of studies centred on dementias started acquiring data in the 2013–2017 timeframe which required a common, harmonized process for MRI data acquisition. This collection defined what is referred to as the Canadian Dementia Imaging Protocol (**CDIP**; www.cdip-pcid.ca)^11^. The CDIP (Fig. 1) includes the following sequences: (a) an isotropic high-resolution 3D T1w MRI; (b) an interleaved PD/T2w MRI; (c) T2* and FLAIR MRIs; (d) a 30+ directions DWI MRI; and (e) a functional connectivity, resting-state BOLD MRI. Parameters for each one of these sequences were harmonized across three MRI vendors, namely General Electric Healthcare, Phillips Medical Systems, and Siemens Healthcare. Sites were evaluated to be CDIP-compliant when they followed a three-step process, namely qualification of the protocol, on-going quality control using geometric and human phantom scans, and on-going quality assurance during the study.

Within this context, SIMON was therefore scanned at 20 different sites in the 2014–2018 time period using this harmonized protocol, namely at the Brain Imaging Center of the Institut universitaire en santé mentale Douglas (Montreal, Canada), the Brain Imaging Center of the Montreal Neurological Institute (Montreal, Canada), the Center for Addiction and Mental Health (Toronto, Canada), the Centre d’Imagerie Moléculaire de Sherbrooke of the Centre Hospitalier Universitaire de Sherbrooke (Sherbrooke, Canada), the Centre Hospitalier Universitaire de Montréal (Montréal, Canada), the Hospital for Sick Children (Toronto, Canada), the IRM Quebec clinic (Quebec City, Canada), the Ottawa Civic Hospital (Ottawa, Canada), the PERFORM Center of Concordia University (Montreal, Canada), the Peter S. Allen MR Research Center of the University of Alberta (Edmonton, Canada), the Robarts Research Institute at the University of Western Ontario (London, Ontario), the Royal University Hospital Radiology Department (Saskatoon, Canada), the Seaman MRI Center of the University of Calgary (Calgary, Canada), the St. Joseph’s Hospital (Hamilton, Canada), the Sunnybrook Hospital (Toronto, Canada), the University of British Columbia MRI Research Center (Vancouver, Canada), the Unité de Neuroimagerie Fonctionnelle du Centre de recherche de l’Institut universitaire en gériatrie de Montréal (Montréal, Canada), the West Coast Medical Imaging Uptown Clinic (Victoria, Canada), and the York University MRI Facility (Toronto, Canada).

### Opportunistic acquisitions - MRIs from various protocols

Over the last 17 years, the same volunteer participated in a number of unrelated academic studies, protocol tests, and manufacturer visits. These scans were all performed within a research, rather than clinical, context and hence are of commensurate quality, albeit with sometimes unique acquisition parameters. Sites visited included the Brain Imaging Center of the Montreal Neurological Institute (Montreal, Canada), the Cuban Neurosciences Center (Havana, Cuba), GE (Milwaukee, WI), IRM Québec (Québec, Canada); the Hôpital Sud (Rennes, France), Philips (Best, The Netherlands), Queen’s University (Kingston, Canada); Siemens (Erlangen, Germany), and St. Michael’s Hospital (Toronto, Canada).

### Image collection and quality control

Since 2007, the MEDICS Laboratory (CERVO Brain Research Centre, Université Laval, Québec, Canada) is responsible for image collection and quality control for all SIMON data, including repatriated scan prior to this time. Further, the MEDICS Laboratory has been responsible since 2013 for all acquisition and analysis including coordination, development and implementation of CDIP at each participating site, site qualification, site quality control via the single volunteer scans, and on-going quality assurance for the CCNA and CIMA-Q studies. The images included in this study were acquired between 2007 and September 2018. The planned data collection includes scans between 2014 and 2018. The opportunistic data collection includes mainly scans between 2001 to 2015, but later scans that derived from the CDIP protocol are also included in this category.

### Abbreviations

Arterial-spin labeling (**ASL**); Consortium pour l’identification précoce de la maladie d’Alzheimer – Québec (**CIMA-Q**); Canadian consortium for neurodegeneration and aging (**CCNA**); Canadian dementia imaging protocol (**CDIP**); Contrast to noise ratio (**CNR**); double inversion recovery (**DIR**); diffusion-weighted imaging (**DWI**); Fluid attenuated inversion recovery (**FLAIR**); Grey matter (**GM**); Magnetic Resonance Imaging (**MRI**); Ontario Brain Institute’s Ontario Neurodegenerative Disease Research Initiative (**ONDRI**); resting state BOLD functional connectivity MRI (**rsfMRI**); Single Individual volunteering for Multiple Observations across Networks (**SIMON**); Susceptibility-weighted imaging (**SWI**); T1-, T2-, T2*, PD-weighted MRI (**T1w**, **T2w**, **PD**, T2*); Time-of-flight (**ToF**); White matter (**WM**).

## Data Records

Characteristics of the MRIs are described in Table 2 for MRIs acquired with the CDIP, and Table 3 for other protocols. The complete dataset is described in Supplementary Table 1 and is publicly available on the Functional Connectomes Project International Neuroimaging Data-Sharing Initiative (INDI) at 10.15387/fcp_indi.retro.simon ^9^. The participating studies are still under recruitment, the requirement for on-going quality control and assurance remains, and new scans of SIMON are likely to be added as time progresses.

**Table 2:** Purposive sample characteristics.

Site	Vendor	Model	Number of sessions*	Time range
CAMH	GE	Discovery MR750	1	2018
CHUM	Philips	Achieva	2	2014–2016
CHUM	Philips	Ingenia	1	2018
CHUS	Philips	Ingenia	3	2014–2016
Cuban Neuroscience Center	Siemens	Allegra	2	2017
Douglas	Siemens	TrioTim	4	2014–2018
Foothills Med. Center U. Calgary	GE	Discovery MR750	2	2016–2018
IUGM	Siemens	TrioTim	3	2014–2016
IUGM	Siemens	Prisma fit	3	2017–2018
McConnell Brain Imaging Centre	Siemens	TrioTim	5	2014–2018
McConnell Brain Imaging Centre	Siemens	Prisma fit	2	2017–2018
Ottawa Hospital Civic	Siemens	TrioTim	1	2018
PERFORM Center	GE	Discovery MR750	2	2015–2017
Peter S. Allen MR Research Center	Siemens	Prisma	2	2016–2018
IRM Quebec	Philips	Achieva	8	2012–2018
Robarts Research Institute	Siemens	Prisma fit	2	2017–2018
Royal University Hospital	Siemens	Skyra	2	2017–2018
Sick Children Hospital	Siemens	Prisma fit	1	2018
St-Joseph Hamilton	GE	Discovery MR750	1	2018
Sunnybrook Research Center	Siemens	Prisma	1	2017
Sunnybrook Research Center	GE	Discovery MR750	1	2018
UBC MRI Research Center	Philips	Intera	2	2016–2018
WCMI Uptown	GE	SIGNA Pioneer	1	2018
York MRI Facility	Siemens	Prisma fit	1	2018

*Some sessions include scan-rescan acquisitions, i.e. images were taken of the volunteer successively, without repositioning.

CAMH: Center for Addiction and Mental Health; CHUM: Centre hospitalier universitaire de Montreal. CHUS: Centre hospitalier universitaire de Sherbrooke; Douglas: Brain Imaging Center of the Institut universitaire en santé mentale Douglas; IUGM: Institut universitaire de gériatrie de Montréal; PERFORM: PERFORM Center, Concordia University; WCMI: West Coast Medical Imaging.

**Table 3:** Opportunistic sample characteristics.

Site	Vendor	Model	Number of sessions	Time range	Acquisitions
Erlangen, GER	Siemens	Prisma	2	2015	T1w; T2w/PD; FLAIR; T2*; DWI; rsfMRI; SWI; ASL
	Siemens	Skyra	1	2015	T1w; T2w/PD; FLAIR; T2*; DWI; rsfMRI; SWI; ASL
Hopital Sud Rennes	Siemens	Symphony	1	2006	T1w; T2w/PD; DWI
McConnell Brain Imaging Centre	Siemens	TrioTim	1	2015	T1w; T2w/PD; FLAIR; T2*; DI; rsfMRI
	Philips	T5	3	2001	T1w
Milwaukee, WI	GE	Discovery MR750	1	2015	T1w; T2w/PD; FLAIR; T1-Cube; T2-Cube; DIR; DWI; ASL
Best, Netherlands	Philips	Ingenia CX	1	2015	T1w; T2w/PD; FLAIR; T2*; DWI; rsfMRI; SWI; ASL
IRM Quebec	Philips	Achieva	7	2009–2012	T1w; T2w/PD; FLAIR; DWI; rsfMRI; ToF
Queen’s University	Siemens	TrioTim	1	2018	T1w; T2w/PD; FLAIR; T2*; DWI; rsfMRI
St. Michael’s Hospital	Siemens	Skyra	1	2018	T1w; T2w/PD; FLAIR; T2*; DWI; rsfMRI
WCMI Uptown	GE	SIGNA Pioneer	1	2017	T1w; T2w/PD; FLAIR; T2*; DWI; rsfMRI

## Technical Validation

### Qualitative control

All images have been reviewed for quality and, when applicable, conformance to the CDIP acquisition parameter values. This review verified the following: coverage, presence of artefacts, and adherence to protocol parameters for purposive acquisitions. Three T1w, one T1-Cube, and one T2-Cube images from the GE manufacture are missing small parts of the right portion of the brain. These images were not included in the analyses.

### Quantitative control

For the quantitatively assess image quality, we calculated the contrast to noise ratio (**CNR**) for the single individual volunteer using voxel intensities from raw T1w images with the mean of grey matter (**GM**) and cerebral white matter (**WM**):

$$CNR=\frac{{\rm{(}}GM\,mean-WM\,mean{{\rm{)}}}^{2}}{(GM\,variance+WM\,variance)}$$

**Fig. 2:**
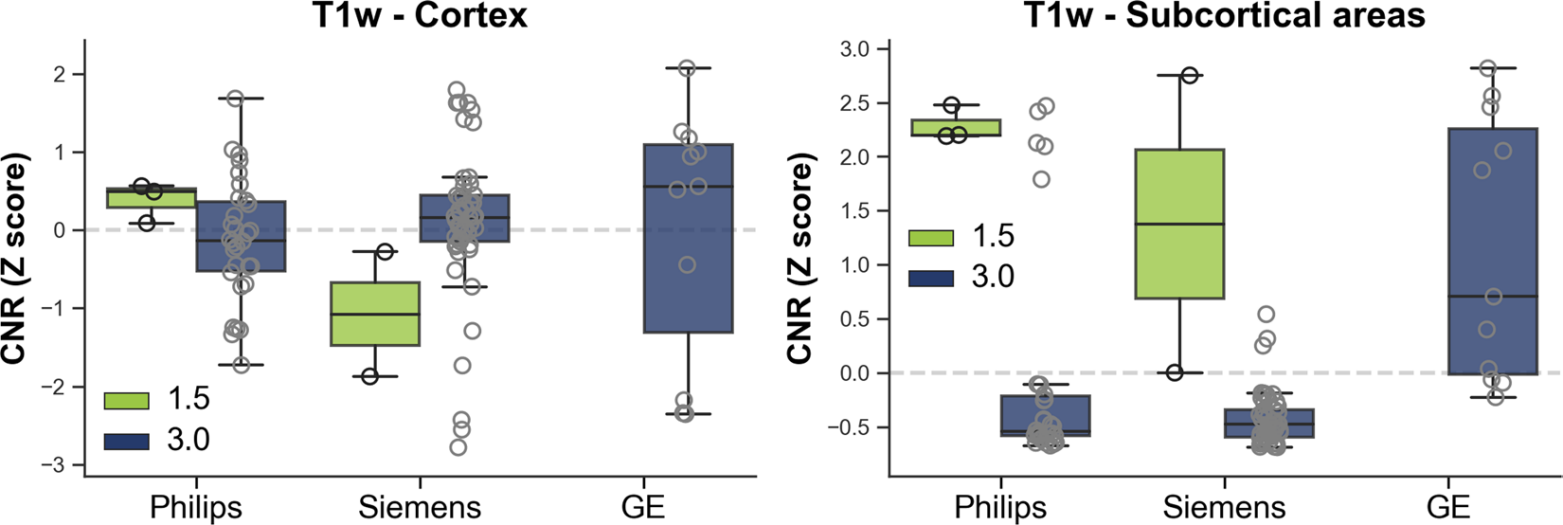
Boxplot illustrating T1w contrast to noise ratio (CNR) for the single individual volunteer across all scans.

This CNR definition was chosen since as it is simple and frequently used^11,12^. Subcortical and cortical GM and WM volumes were obtained from the aparc and aseg labels derived from automated segmentation using *FreeSurfer* (version 5.3 with default parameters; freesurfer.net). Please note that *FreeSurfer* segmentations are not distributed with the dataset. The technical details of *FreeSurfer* procedures are described in prior publications^13–17^. CNR was computed using the uncorrected T1w image (orig.mgz file). Supplementary Table 2 and Fig. 2 shows subcortical and cortical T1w CNR for the single individual volunteer across all scans according to scanner vendor. Kruskal-Wallis tests indicate a significant different CNR for subcortical (*H*: 18.65, *p* < 0.0001, mean ± sd GE: 1.68 ± 1.05, Philips: 0.79 ± 1.07, Siemens: 0.32 ± 0.46), but not for cortical areas (*H*: 1.92, *p* = 0.3837, GE: 3.18 ± 1.61, Philips: 3.07 ± 0.80, Siemens: 3.21 ± 1.04) across vendor. Mann-Whitney tests revealed that GE had higher subcortical CNR than Philips (*U*: 67, *p* = 0.0010) and Siemens (*U*: 30, *p* < 0.0001) scanners.

**Fig. 3:**
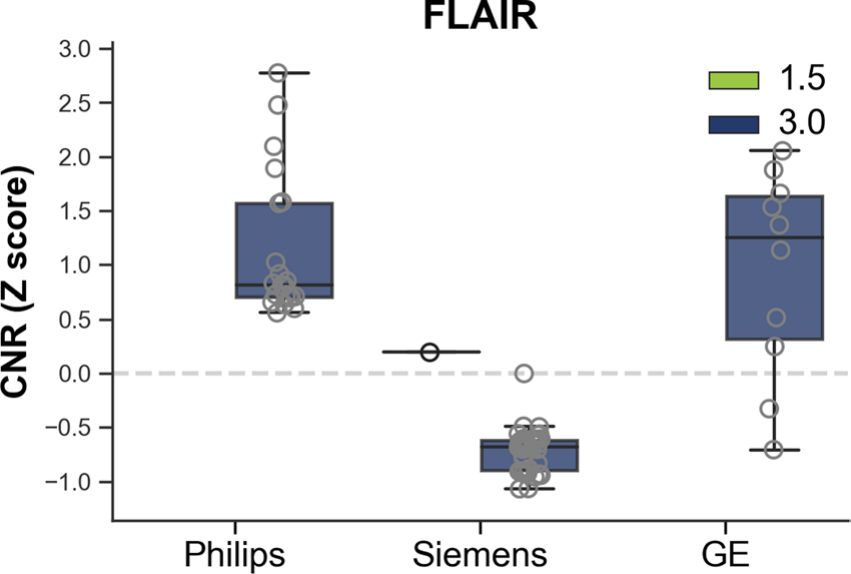
Boxplot illustrating FLAIR contrast to noise ratio (CNR) for the single individual volunteer across all scans.

In order to compute CNR for FLAIR images, the same equation as T1w was used. However, since there is little contrast between WM and GM classes in FLAIR, the first tissue class is called the brain class (which is GM + WM as a whole), and the second tissue class is the CSF. In order to compute the mean and standard deviations for the brain and CSF tissues, segmentations of these tissues were required. Tissue segmentation was generated using a standardization and segmentation framework for FLAIR MRI^18–20^. To ensure that the tissue classes contained pure tissues only, 70% of the middle slices were retained for CNR calculation, ensuring that if the brain extraction algorithm missed any skull at the top or bottom, it would not bias the approach. Results are shown in Supplementary Table 3 and Fig. 3 and show that FLAIR CNR significantly differs between vendors (*H*: 50.83, *p* < 0.0001, GE: 346.17 ± 0.00, Philips: 1270.08 ± 326.33, Siemens: 340.95 ± 102.71), with Siemens having lower CNR than Philips (*U*: 0, *p* < 0.0001) and GE (*U*: 32, *p* < 0.0001) manufacturers.

**Fig. 4:**
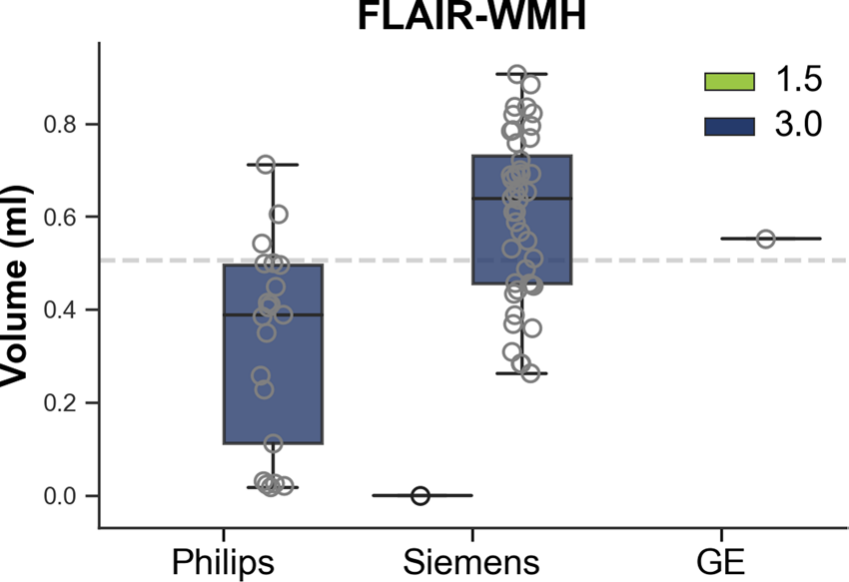
Boxplot illustrating FLAIR white matter hyperintensities (WMH) for the single individual volunteer across all scans.

Although, the participant is a healthy individual, white matter hyperintensity (WMH) on FLAIR was assessed using Schmidt *et al*.’s automated VBM toolbox^21^ (GE: 0.55 ml ± 0.00, Philips: 0.33 ml ± 0.21, Siemens: 0.59 ml ± 0.19). Figure 4 shows that the volume of WMH was larger (*U*:, 177, *p* < 0.0001) on Siemens than on Philips scanners (see all results in Supplementary Table 2).

**Fig. 5:**
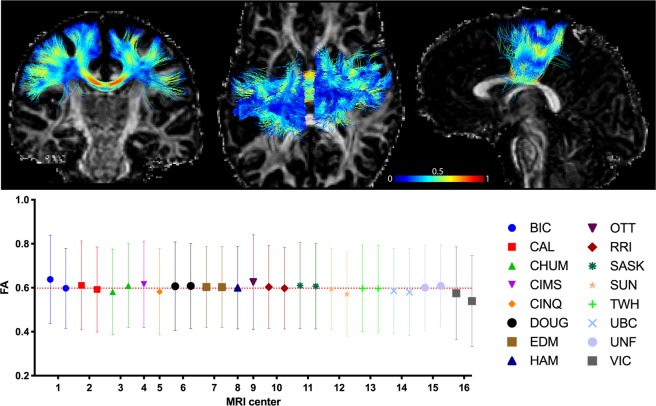
Whole-brain tractography according to multiple scan sites (MRI center). Top: Example of a tract section located in the mid-body of the corpus callosum, color coded for fractional anisotropy (FA) value. Bottom: Mean FA value average across all voxels within the tract. Errors bars represent standard deviations.

Whole-brain tractography was done using the TractoFlow pipeline^22,23^. Figure 5 displays the mean fractional anisotropy (FA) of a tract section located in the mid-body of the corpus callosum. Based on 28 DWI scans from 16 different scanners, this pipeline is dividing brain white matter into 12 bilateral and 9 commissural tracts. The mean FA value obtained from this tract displays high agreement and shows large SD as voxels located in the central core of the corpus callosum have FA values ~0.8 while voxels near the end of the fibers and at crossing fibers have lower FA values.

**Fig. 6:**
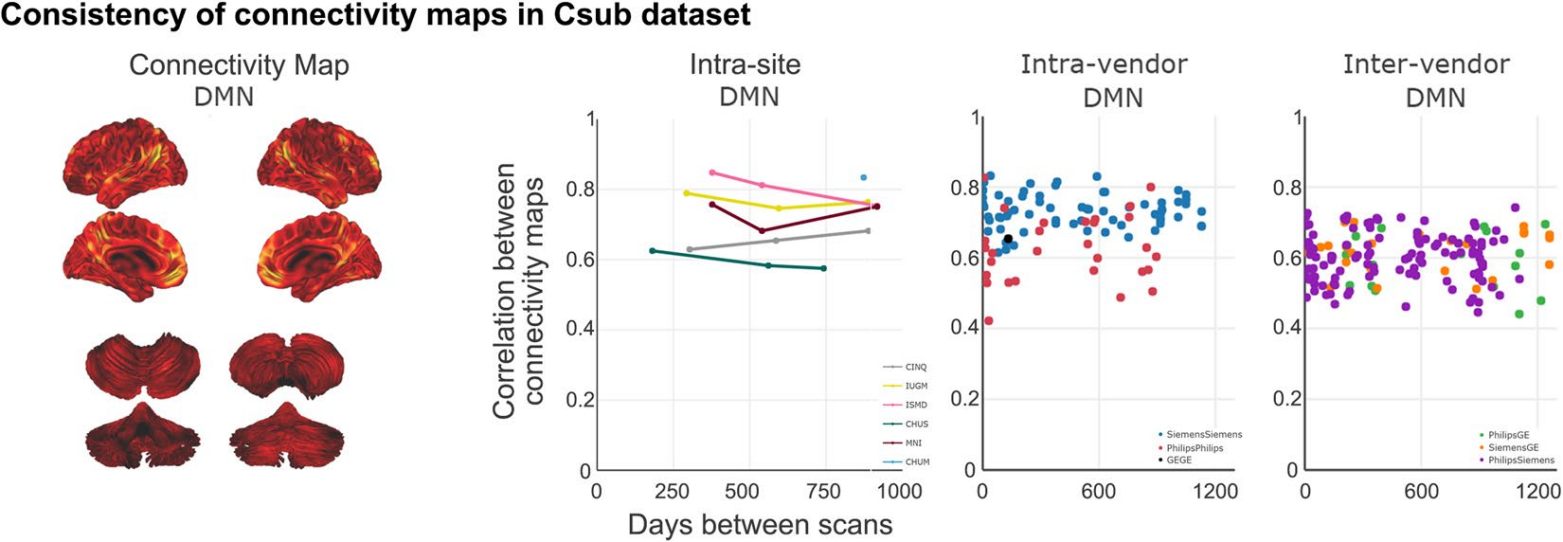
Default mode network (DMN) average of all 24 scans, along with intra-site, intra-vendor and inter-vendor consistency of DMN connectivity over time. For the connectivity map brighter colours (orange-yellow) in the connectivity maps indicate stronger connectivity strength. Map is superimposed onto the anatomic International Consortium for Brain Mapping (ICBM) 152 template and the SPM2_MNI aligned cerebellum surface^27^.

Stability of resting-state networks across time and vendors were assessed using 24 scans (from the planned data collection), acquired at 13 sites, on three scanner vendors^24^. In brief, to assess the stability of resting-state networks, voxel-wise connectivity maps associated from seven MIST atlas network templates^25^ were computed for each rsfMRI scan using NIAK’s connectome pipeline^26^ (http://niak.simexp-lab.org/pipe_connectome.html). For each of the seven rsfMRI networks, a scan by scan similarity (Pearson’s correlation) matrix was generated to summarize the consistency of connectivity maps across the 24 scans. Figure 6 shows the average connectivity for the default-mode network (DMN) for the 24 scans, along with the intra-site (for the six sites with 2 or more scans), intra-vendor and inter-vendor consistency of DMN connectivity over time. Moreover, a series of explanatory variables (time between scans, intra-vendor, inter-vendor, intercept) were assembled for a general linear model analysis. A linear mixture of the explanatory variables were adjusted on the inter-scan consistency measures (dependent variable) using ordinary least squares, for each network separately. For each network, the significance of the effect of inter-vendor, intra-vendor, intra-site and time were tested.

## Usage Notes

This dataset^9^, composed of a single individual volunteer data, is useful for testing cross-sectional and longitudinal stability of image processing algorithms across scanner models and vendors. To assess variability across sites in multi-centric studies, such data are required and are relatively rare, and their availability is of paramount importance to the field of image processing. Researchers can compare variance within and between techniques, homogeneity, influence of parameters on final measurements, or the effect of various corrective techniques. While most scans are relatively recent, one should note that images in the dataset were acquired when the subject was between 28 to 46 years old and thus, age need to be taken into account for measurement reliability analyses.

The CDIP protocol is already in use as the core protocol for the CIMA-Q study (143 participants; seven sites), and the *Ontario Brain Institute’s Ontario Neurodegenerative Disease Research Initiative* (~600 participants, 11 sites). It serves as the basis for the core protocol of the *Canadian Alliance for Health Hearts and Minds*, whereas the core protocol (~7,000 participants) includes both T1-weighted and FLAIR acquisitions, and the extended version is the complete CDIP (~1,500 participants; eight sites). It has been deployed at more than 19 sites in Canada as part of the CCNA (~1,600 participants). Together, these studies plan on recruiting well over 10,000 participants, across adulthood and a range of cognitive conditions. The protocol has now reached beyond Canadian shores and is being used at the Human Brain Mapping Unit (Cuban Neuroscience Center, Habana, Cuba) and the University of Electronic Science and Technology of China (Chengdu, China).

A website has been established (www.cdip-pcid.ca) to collect all information regarding the protocol. Dissemination is free and only requires acknowledging the current effort. On the site, visitors can find (a) the full protocol, with all harmonized parameters detailed for each vendor; (b) exam cards or PDF output for all tested scanners to date from the three major vendors, ready for upload in a similar machine; (c) a complete operator manual for MR technologists, complete with descriptions of the procedure to scan the geometric phantom, the single individual volunteer, and any study participant, with a list of common acquisition artefacts and pointers to correct them; and (d) the SIMON datasets.

## Supplementary information

Supplementary Table 1

Supplementary Table 2

Supplementary Table 3

## Data Availability

No custom code was used to produce the dataset.
