# Supplementary material for: Structural and functional multi-platform MRI series of a single human volunteer over more than fifteen years
Source: Sci Data. 2019 Oct 31;6:245. doi: 10.1038/s41597-019-0262-8 (PMC6823440; doi:10.1038/s41597-019-0262-8)
Supplement: Supplementary file 1 — Supplementary Table 1 [file 41597_2019_262_MOESM1_ESM.docx]

Supplementary Table 1. Description of each MRI.

| Session | Modality | Manufacturer | Model | Site | Acquisition Date Time | Magnetic Field Strength | Slice Thickness | TE | TR | Flip Angle |
| --- | --- | --- | --- | --- | --- | --- | --- | --- | --- | --- |
| 1 | T1w | Philips | T5 | MONTREAL NEURO | 2001-09-20T08:00:19 | 1.5 | 1 | 0.009992779 | 0.018049 | 30 |
| 2 | T1w | Philips | T5 | Montreal Neuro | 2002-06-17T12:58:41 | 1.5 | 1 | 0.01008878 | 0.018049 | 30 |
| 3 | T1w | Philips | T5 | Montreal Neuro | 2004-03-18T11:52:56 | 1.5 | 1 | 0.01012478 | 0.018049 | 30 |
| 4 | PD | Siemens | Symphony | HOPITAL_SUD_RENNES | 2006-07-21T13:03:41.940000 | 1.5 | 3 | 0.11 | 5.84 | 180 |
| 4 | T1w | Siemens | Symphony | HOPITAL_SUD_RENNES | 2006-07-21T13:13:3.465020 | 1.5 | 1 | 0.0051 | 0.015 | 30 |
| 4 | T2w | Siemens | Symphony | HOPITAL_SUD_RENNES | 2006-07-21T13:03:41.880010 | 1.5 | 3 | 0.012 | 5.84 | 180 |
| 4 | DWI | Siemens | Symphony | HOPITAL_SUD_RENNES | 2006-07-21T13:53:45.949980 | 1.5 | 5 | 0.114 | 4 | 90 |
| 4 | DWI | Siemens | Symphony | HOPITAL_SUD_RENNES | 2006-07-21T13:56:57.925000 | 1.5 | 5 | 0.114 | 4 | 90 |
| 4 | DWI | Siemens | Symphony | HOPITAL_SUD_RENNES | 2006-07-21T14:00:10.929990 | 1.5 | 5 | 0.114 | 4 | 90 |
| 4 | DWI | Siemens | Symphony | HOPITAL_SUD_RENNES | 2006-07-21T13:24:47.857500 | 1.5 | 5 | 0.114 | 4 | 90 |
| 4 | DWI | Siemens | Symphony | HOPITAL_SUD_RENNES | 2006-07-21T13:28:0.932490 | 1.5 | 5 | 0.114 | 4 | 90 |
| 4 | DWI | Siemens | Symphony | HOPITAL_SUD_RENNES | 2006-07-21T13:31:13.940010 | 1.5 | 5 | 0.114 | 4 | 90 |
| 4 | DWI | Siemens | Symphony | HOPITAL_SUD_RENNES | 2006-07-21T13:34:27.000000 | 1.5 | 5 | 0.114 | 4 | 90 |
| 4 | DWI | Siemens | Symphony | HOPITAL_SUD_RENNES | 2006-07-21T13:37:39.922510 | 1.5 | 5 | 0.114 | 4 | 90 |
| 4 | DWI | Siemens | Symphony | HOPITAL_SUD_RENNES | 2006-07-21T13:40:53.004990 | 1.5 | 5 | 0.114 | 4 | 90 |
| 4 | DWI | Siemens | Symphony | HOPITAL_SUD_RENNES | 2006-07-21T13:44:5.947490 | 1.5 | 5 | 0.114 | 4 | 90 |
| 4 | DWI | Siemens | Symphony | HOPITAL_SUD_RENNES | 2006-07-21T13:47:18.914980 | 1.5 | 5 | 0.114 | 4 | 90 |
| 4 | DWI | Siemens | Symphony | HOPITAL_SUD_RENNES | 2006-07-21T13:50:31.900020 | 1.5 | 5 | 0.114 | 4 | 90 |
| 5 | T1w | Philips | Achieva | IRM_Quebec_-_Mailloux_-_3T | 2009-09-17T10:08:0.040000 | 3 | 1 | 0.003716 | 0.008124 | 8 |
| 5 | T2w | Philips | Achieva | IRM_Quebec_-_Mailloux_-_3T | 2009-09-17T10:22:48.930000 | 3 | 4 | 0.08 | 3 | 90 |
| 5 | T1w | Philips | Achieva | IRM_Quebec_-_Mailloux_-_3T | 2009-09-17T10:16:1.820000 | 3 | 1 | 0.003807 | 0.0083029 | 8 |
| 5 | T2w | Philips | Achieva | IRM_Quebec_-_Mailloux_-_3T | 2009-09-17T10:25:0.590000 | 3 | 1 | 0.381605 | 2.5 | 90 |
| 5 | T1w | Philips | Achieva | IRM_Quebec_-_Mailloux_-_3T | 2009-09-17T10:58:54.070000 | 3 | 1 | 0.003716 | 0.008124 | 8 |
| 5 | DWI | Philips | Achieva | IRM_Quebec_-_Mailloux_-_3T | 2009-09-17T11:06:33.540000 | 3 | 2 | 0.07 | 7.90437 | 90 |
| 5 | rsfMRI | Philips | Achieva | IRM_Quebec_-_Mailloux_-_3T | 2009-09-17T11:16:53.120000 | 3 | 4 | 0.035 | 3 | 90 |
| 6 | PD | Philips | Achieva | IRM_Quebec_-_Mailloux_-_3T | 2009-12-11T14:42:34.980000 | 3 | 2 | 0.008 | 3 | 90 |
| 6 | T1w | Philips | Achieva | IRM_Quebec_-_Mailloux_-_3T | 2009-12-11T14:29:13.370000 | 3 | 1 | 0.0031348 | 0.0069222 | 8 |
| 6 | T2w | Philips | Achieva | IRM_Quebec_-_Mailloux_-_3T | 2009-12-11T14:42:34.980000 | 3 | 2 | 0.1 | 3 | 90 |
| 6 | T1w | Philips | Achieva | IRM_Quebec_-_Mailloux_-_3T | 2009-12-11T14:35:57.710000 | 3 | 1 | 0.0031348 | 0.0069222 | 8 |
| 7 | T1w | Philips | Achieva | IRM_Quebec_-_Mailloux_-_3T | 2010-03-19T14:54:13.480000 | 3 | 1 | 0.0037286 | 0.0081471 | 8 |
| 7 | DWI | Philips | Achieva | IRM_Quebec_-_Mailloux_-_3T | 2010-03-19T15:02:26.000000 | 3 | 1.8 | 0.0650133 | 7.40275 | 90 |
| 7 | DWI | Philips | Achieva | IRM_Quebec_-_Mailloux_-_3T | 2010-03-19T15:05:58.900000 | 3 | 1.8 | 0.065125 | 8.90089 | 90 |
| 8 | T1w | Philips | Achieva | IRM_Quebec_-_Mailloux_-_3T | 2010-09-10T13:58:10 | 3 |  |  |  |  |
| 8 | T2w | Philips | Achieva | IRM_Quebec_-_Mailloux_-_3T | 2010-09-10T13:58:10 | 3 |  |  |  |  |
| 9 | FLAIR | Philips | Achieva | IRM_Quebec_-_Mailloux_-_3T | 2010-11-03T19:48:34.280000 | 3 | 1.5 | 0.125 | 11 | 90 |
| 9 | PD | Philips | Achieva | IRM_Quebec_-_Mailloux_-_3T | 2010-11-03T19:36:21.560000 | 3 | 1.5 | 0.008 | 3 | 90 |
| 9 | T1w | Philips | Achieva | IRM_Quebec_-_Mailloux_-_3T | 2010-11-03T19:29:35.140000 | 3 | 1 | 0.0031399 | 0.0068997 | 8 |
| 9 | T2w | Philips | Achieva | IRM_Quebec_-_Mailloux_-_3T | 2010-11-03T19:36:21.560000 | 3 | 1.5 | 0.1 | 3 | 90 |
| 10 | PD | Philips | Achieva | IRM_Quebec_-_Mailloux_-_3T | 2012-08-20T15:02:16.140000 | 3 | 1.5 | 0.008 | 3 | 90 |
| 10 | T1w | Philips | Achieva | IRM_Quebec_-_Mailloux_-_3T | 2012-08-20T14:55:29.090000 | 3 | 1 | 0.003137 | 0.0068843 | 8 |
| 10 | T2w | Philips | Achieva | IRM_Quebec_-_Mailloux_-_3T | 2012-08-20T15:02:16.140000 | 3 | 1.5 | 0.1 | 3 | 90 |
| 11 | FLAIR | Siemens | TrioTim | Mc_Gill_University | 2014-02-18T11:31:28.755000 | 3 | 3 | 0.123 | 9 | 165 |
| 11 | PD | Siemens | TrioTim | Mc_Gill_University | 2014-02-18T11:16:27.747500 | 3 | 3 | 0.01 | 3 | 165 |
| 11 | T1w | Siemens | TrioTim | Mc_Gill_University | 2014-02-18T11:00:43.522500 | 3 | 1 | 0.00298 | 2.3 | 9 |
| 11 | T2star | Siemens | TrioTim | Mc_Gill_University | 2014-02-18T11:45:1.227500 | 3 | 3 | 0.02 | 0.65 | 20 |
| 11 | T2w | Siemens | TrioTim | Mc_Gill_University | 2014-02-18T11:16:27.840000 | 3 | 3 | 0.091 | 3 | 165 |
| 11 | FLAIR | Siemens | TrioTim | Mc_Gill_University | 2014-02-18T11:38:16.555000 | 3 | 3 | 0.123 | 9 | 165 |
| 11 | PD | Siemens | TrioTim | Mc_Gill_University | 2014-02-18T11:21:47.140000 | 3 | 3 | 0.01 | 3 | 165 |
| 11 | T1w | Siemens | TrioTim | Mc_Gill_University | 2014-02-18T11:10:45.600000 | 3 | 1 | 0.00298 | 2.3 | 9 |
| 11 | T2star | Siemens | TrioTim | Mc_Gill_University | 2014-02-18T11:49:21.580000 | 3 | 3 | 0.02 | 0.65 | 20 |
| 11 | T2w | Siemens | TrioTim | Mc_Gill_University | 2014-02-18T11:21:47.212500 | 3 | 3 | 0.091 | 3 | 165 |
| 11 | DWI | Siemens | TrioTim | Mc_Gill_University | 2014-02-18T12:00:11.347500 | 3 | 2 | 0.096 | 9.4 | 90 |
| 11 | DWI | Siemens | TrioTim | Mc_Gill_University | 2014-02-18T12:05:30.227500 | 3 | 2 | 0.1307 | 13.219 | 90 |
| 11 | rsfMRI | Siemens | TrioTim | Mc_Gill_University | 2014-02-18T12:15:34.937500 | 3 | 3.5 | 0.03 | 2.11 | 70 |
| 11 | rsfMRI | Siemens | TrioTim | Mc_Gill_University | 2014-02-18T12:26:32.052500 | 3 | 3.5 | 0.03 | 2.38 | 70 |
| 12 | FLAIR | Philips | Achieva | IRM_Quebec_-_Mailloux_-_3T | 2014-02-25T11:25:32.930000 | 3 | 3 | 0.09 | 9 | 90 |
| 12 | PD | Philips | Achieva | IRM_Quebec_-_Mailloux_-_3T | 2014-02-25T11:06:10.370000 | 3 | 3 | 0.010526 | 2.5 | 90 |
| 12 | T1w | Philips | Achieva | IRM_Quebec_-_Mailloux_-_3T | 2014-02-25T10:52:34.260000 | 3 | 1 | 0.003136 | 0.0069285 | 9 |
| 12 | T2star | Philips | Achieva | IRM_Quebec_-_Mailloux_-_3T | 2014-02-25T11:10:54.980000 | 3 | 3 | 0.020721 | 0.65 | 20 |
| 12 | T2w | Philips | Achieva | IRM_Quebec_-_Mailloux_-_3T | 2014-02-25T11:06:10.370000 | 3 | 3 | 0.1 | 2.5 | 90 |
| 12 | DWI | Philips | Achieva | IRM_Quebec_-_Mailloux_-_3T | 2014-02-25T11:15:27.480000 | 3 | 2 | 0.065125 | 6 | 90 |
| 13 | FLAIR | Philips | Achieva | IRM_Quebec_-_Mailloux_-_3T | 2014-03-14T10:45:18.100000 | 3 | 3 | 0.09 | 9 | 90 |
| 13 | PD | Philips | Achieva | IRM_Quebec_-_Mailloux_-_3T | 2014-03-14T10:40:33.480000 | 3 | 3 | 0.010526 | 2.5 | 90 |
| 13 | T1w | Philips | Achieva | IRM_Quebec_-_Mailloux_-_3T | 2014-03-14T10:58:22.310000 | 3 | 1 | 0.003333 | 0.0073312 | 9 |
| 13 | T2star | Philips | Achieva | IRM_Quebec_-_Mailloux_-_3T | 2014-03-14T10:53:51.320000 | 3 | 3 | 0.020721 | 0.65 | 20 |
| 13 | T2w | Philips | Achieva | IRM_Quebec_-_Mailloux_-_3T | 2014-03-14T10:40:33.480000 | 3 | 3 | 0.1 | 2.5 | 90 |
| 13 | DWI | Philips | Achieva | IRM_Quebec_-_Mailloux_-_3T | 2014-03-14T11:22:55.310000 | 3 | 2 | 0.100741 | 10.0286 | 90 |
| 13 | rsfMRI | Philips | Achieva | IRM_Quebec_-_Mailloux_-_3T |  | 3 | 3.5 | 0.030001 | 2.11 | 70 |
| 14 | FLAIR | Siemens | TrioTim | Mc_Gill_University | 2014-05-20T10:13:34.170000 | 3 | 3 | 0.123 | 9 | 165 |
| 14 | PD | Siemens | TrioTim | Mc_Gill_University | 2014-05-20T10:07:59.092500 | 3 | 3 | 0.01 | 3 | 165 |
| 14 | T1w | Siemens | TrioTim | Mc_Gill_University | 2014-05-20T10:01:41.462500 | 3 | 1 | 0.00298 | 2.3 | 9 |
| 14 | T2star | Siemens | TrioTim | Mc_Gill_University | 2014-05-20T10:19:22.867500 | 3 | 3 | 0.02 | 0.65 | 20 |
| 14 | T2w | Siemens | TrioTim | Mc_Gill_University | 2014-05-20T10:07:59.185000 | 3 | 3 | 0.091 | 3 | 165 |
| 14 | DWI | Siemens | TrioTim | Mc_Gill_University | 2014-05-20T10:21:35.355000 | 3 | 2 | 0.096 | 9.4 | 90 |
| 14 | rsfMRI | Siemens | TrioTim | Mc_Gill_University | 2014-05-20T10:28:54.522500 | 3 | 3.5 | 0.03 | 2.11 | 70 |
| 15 | FLAIR | Siemens | TrioTim | Hospital_Douglas | 2014-05-20T16:38:53.827500 | 3 | 3 | 0.123 | 9 | 165 |
| 15 | PD | Siemens | TrioTim | Hospital_Douglas | 2014-05-20T16:33:10.542500 | 3 | 3 | 0.01 | 3 | 165 |
| 15 | T1w | Siemens | TrioTim | Hospital_Douglas | 2014-05-20T16:27:31.797500 | 3 | 1 | 0.00298 | 2.3 | 9 |
| 15 | T2star | Siemens | TrioTim | Hospital_Douglas | 2014-05-20T16:44:25.072500 | 3 | 3 | 0.02 | 0.65 | 20 |
| 15 | T2w | Siemens | TrioTim | Hospital_Douglas | 2014-05-20T16:33:10.635000 | 3 | 3 | 0.091 | 3 | 165 |
| 15 | DWI | Siemens | TrioTim | Hospital_Douglas | 2014-05-20T16:46:41.480000 | 3 | 2 | 0.096 | 9.4 | 90 |
| 15 | rsfMRI | Siemens | TrioTim | Hospital_Douglas | 2014-05-20T16:53:46.855000 | 3 | 3.5 | 0.03 | 2.11 | 70 |
| 16 | FLAIR | Siemens | TrioTim | IUGM | 2014-05-21T11:36:37.900000 | 3 | 3 | 0.123 | 9 | 165 |
| 16 | PD | Siemens | TrioTim | IUGM | 2014-05-21T11:31:3.227500 | 3 | 3 | 0.01 | 3 | 165 |
| 16 | T1w | Siemens | TrioTim | IUGM | 2014-05-21T11:24:43.477500 | 3 | 1 | 0.00298 | 2.3 | 9 |
| 16 | T2star | Siemens | TrioTim | IUGM | 2014-05-21T11:42:31.330000 | 3 | 3 | 0.02 | 0.65 | 20 |
| 16 | T2w | Siemens | TrioTim | IUGM | 2014-05-21T11:31:3.320000 | 3 | 3 | 0.091 | 3 | 165 |
| 16 | DWI | Siemens | TrioTim | IUGM | 2014-05-21T11:45:4.367500 | 3 | 2 | 0.096 | 9.4 | 90 |
| 16 | rsfMRI | Siemens | TrioTim | IUGM | 2014-05-21T11:52:28.662500 | 3 | 3.5 | 0.03 | 2.11 | 70 |
| 17 | FLAIR | Philips | Achieva | IRM_Quebec_-_Mailloux_-_3T | 2014-06-11T09:45:43.560000 | 3 | 3 | 0.125 | 9 | 90 |
| 17 | PD | Philips | Achieva | IRM_Quebec_-_Mailloux_-_3T | 2014-06-11T09:40:59.340000 | 3 | 3 | 0.010526 | 2.5 | 90 |
| 17 | T1w | Philips | Achieva | IRM_Quebec_-_Mailloux_-_3T | 2014-06-11T09:33:46.350000 | 3 | 1 | 0.00331 | 0.007304 | 9 |
| 17 | T2star | Philips | Achieva | IRM_Quebec_-_Mailloux_-_3T | 2014-06-11T09:50:40.280000 | 3 | 3 | 0.020721 | 0.65 | 20 |
| 17 | T2w | Philips | Achieva | IRM_Quebec_-_Mailloux_-_3T | 2014-06-11T09:40:59.340000 | 3 | 3 | 0.1 | 2.5 | 90 |
| 17 | DWI | Philips | Achieva | IRM_Quebec_-_Mailloux_-_3T | 2014-06-11T09:55:11.670000 | 3 | 2 | 0.100741 | 10.002 | 90 |
| 17 | rsfMRI | Philips | Achieva | IRM_Quebec_-_Mailloux_-_3T | 2014-06-11T10:01:36.260000 | 3 | 3.5 | 0.030001 | 2.11 | 70 |
| 18 | FLAIR | Philips | Achieva | CHUM_Campus_Notre-Dame | 2014-07-03T17:05:14.060000 | 3 | 3 | 0.125 | 9 | 90 |
| 18 | PD | Philips | Achieva | CHUM_Campus_Notre-Dame | 2014-07-03T17:00:45.480000 | 3 | 3 | 0.0125 | 3 | 90 |
| 18 | T1w | Philips | Achieva | CHUM_Campus_Notre-Dame | 2014-07-03T16:53:57.530000 | 3 | 1 | 0.003296 | 0.0072777 | 9 |
| 18 | T2star | Philips | Achieva | CHUM_Campus_Notre-Dame | 2014-07-03T17:10:11.250000 | 3 | 3 | 0.020716 | 0.65 | 20 |
| 18 | T2w | Philips | Achieva | CHUM_Campus_Notre-Dame | 2014-07-03T17:00:45.480000 | 3 | 3 | 0.1 | 3 | 90 |
| 18 | DWI | Philips | Achieva | CHUM_Campus_Notre-Dame | 2014-07-03T17:14:56.460000 | 3 | 2 | 0.100459 | 9.94539 | 90 |
| 18 | rsfMRI | Philips | Achieva | CHUM_Campus_Notre-Dame | 2014-07-03T17:21:28.510000 | 3 | 3.5 | 0.030001 | 2.11 | 70 |
| 19 | FLAIR | Philips | Achieva | IRM_Quebec_-_Mailloux_-_3T | 2014-09-19T09:46:16.710000 | 3 | 3 | 0.09 | 9 | 90 |
| 19 | PD | Philips | Achieva | IRM_Quebec_-_Mailloux_-_3T | 2014-09-19T09:41:27.100000 | 3 | 3 | 0.010526 | 2.5 | 90 |
| 19 | T1w | Philips | Achieva | IRM_Quebec_-_Mailloux_-_3T | 2014-09-19T10:00:7.700000 | 3 | 1 | 0.003363 | 0.0073585 | 9 |
| 19 | T2star | Philips | Achieva | IRM_Quebec_-_Mailloux_-_3T | 2014-09-19T09:54:49.560000 | 3 | 3 | 0.02072 | 0.65 | 20 |
| 19 | T2w | Philips | Achieva | IRM_Quebec_-_Mailloux_-_3T | 2014-09-19T09:41:27.100000 | 3 | 3 | 0.1 | 2.5 | 90 |
| 19 | rsfMRI | Philips | Achieva | IRM_Quebec_-_Mailloux_-_3T | 2014-09-19T10:06:40.510000 | 3 | 3.5 | 0.03 | 2.11 | 70 |
| 20 | FLAIR | Philips | Achieva | IRM_Quebec_-_Mailloux_-_3T | 2014-09-24T08:40:53.560000 | 3 | 3 | 0.09 | 9 | 90 |
| 20 | PD | Philips | Achieva | IRM_Quebec_-_Mailloux_-_3T | 2014-09-24T08:36:9.420000 | 3 | 3 | 0.010526 | 2.5 | 90 |
| 20 | T1w | Philips | Achieva | IRM_Quebec_-_Mailloux_-_3T | 2014-09-24T08:18:12.340000 | 3 | 1 | 0.003338 | 0.007361 | 9 |
| 20 | T2star | Philips | Achieva | IRM_Quebec_-_Mailloux_-_3T | 2014-09-24T08:49:26.370000 | 3 | 3 | 0.02072 | 0.65 | 20 |
| 20 | T2w | Philips | Achieva | IRM_Quebec_-_Mailloux_-_3T | 2014-09-24T08:36:9.420000 | 3 | 3 | 0.1 | 2.5 | 90 |
| 20 | DWI | Philips | Achieva | IRM_Quebec_-_Mailloux_-_3T | 2014-09-24T08:53:59.730000 | 3 | 2 | 0.1024 | 10.1085 | 90 |
| 20 | rsfMRI | Philips | Achieva | IRM_Quebec_-_Mailloux_-_3T | 2014-09-24T08:24:58.340000 | 3 | 3.5 | 0.03 | 2.11 | 70 |
| 21 | FLAIR | Philips | Achieva | IRM_Quebec_-_Mailloux_-_3T | 2014-09-24T14:23:39.480000 | 3 | 3 | 0.09 | 9 | 90 |
| 21 | PD | Philips | Achieva | IRM_Quebec_-_Mailloux_-_3T | 2014-09-24T14:18:55.230000 | 3 | 3 | 0.010526 | 2.5 | 90 |
| 21 | T1w | Philips | Achieva | IRM_Quebec_-_Mailloux_-_3T | 2014-09-24T14:00:56.700000 | 3 | 1 | 0.003345 | 0.0073766 | 9 |
| 21 | T2star | Philips | Achieva | IRM_Quebec_-_Mailloux_-_3T | 2014-09-24T14:32:12.340000 | 3 | 3 | 0.02072 | 0.65 | 20 |
| 21 | T2w | Philips | Achieva | IRM_Quebec_-_Mailloux_-_3T | 2014-09-24T14:18:55.230000 | 3 | 3 | 0.1 | 2.5 | 90 |
| 21 | DWI | Philips | Achieva | IRM_Quebec_-_Mailloux_-_3T | 2014-09-24T14:36:44.670000 | 3 | 2 | 0.100741 | 9.99622 | 90 |
| 21 | rsfMRI | Philips | Achieva | IRM_Quebec_-_Mailloux_-_3T | 2014-09-24T14:07:42.620000 | 3 | 3.5 | 0.03 | 2.11 | 70 |
| 22 | FLAIR | Philips | Ingenia | CHUS_FLEURIMONT_Philips_3t | 2014-10-23T15:25:28.680000 | 3 | 3 | 0.125 | 9 | 90 |
| 22 | PD | Philips | Ingenia | CHUS_FLEURIMONT_Philips_3t | 2014-10-23T15:19:59.800000 | 3 | 3 | 0.0125 | 3 | 90 |
| 22 | T1w | Philips | Ingenia | CHUS_FLEURIMONT_Philips_3t | 2014-10-23T15:13:26.090000 | 3 | 1 | 0.003299 | 0.0072922 | 9 |
| 22 | T2star | Philips | Ingenia | CHUS_FLEURIMONT_Philips_3t | 2014-10-23T15:29:1.560000 | 3 | 3 | 0.020719 | 0.65 | 20 |
| 22 | T2w | Philips | Ingenia | CHUS_FLEURIMONT_Philips_3t | 2014-10-23T15:19:59.800000 | 3 | 3 | 0.1 | 3 | 90 |
| 22 | DWI | Philips | Ingenia | CHUS_FLEURIMONT_Philips_3t | 2014-10-23T15:45:17.530000 | 3 | 2 | 0.106946 | 9.97324 | 90 |
| 22 | rsfMRI | Philips | Ingenia | CHUS_FLEURIMONT_Philips_3t | 2014-10-23T15:34:8.980000 | 3 | 3.5 | 0.03 | 2.11 | 70 |
| 23 | FLAIR | Siemens | TrioTim | IUGM | 2015-03-10T10:40:08.215000 | 3 | 3 | 0.123 | 9 | 165 |
| 23 | PD | Siemens | TrioTim | IUGM | 2015-03-10T10:34:34.212500 | 3 | 3 | 10 | 3000 | 165 |
| 23 | T1w | Siemens | TrioTim | IUGM | 2015-03-10T10:28:50.697500 | 3 | 1 | 2.98 | 2300 | 9 |
| 23 | T2star | Siemens | TrioTim | IUGM | 2015-03-10T10:45:45.072500 | 3 | 3 | 20 | 650 | 20 |
| 23 | T2w | Siemens | TrioTim | IUGM | 2015-03-10T10:34:34.302500 | 3 | 3 | 91 | 3000 | 165 |
| 23 | DWI | Siemens | TrioTim | IUGM | 2015-03-10T10:52:07.625 | 3 | 2 | 96 | 9400 | 90 |
| 23 | rsfMRI | Siemens | TrioTim | IUGM | 2015-03-10T10:56:59.980000 | 3 | 3.5 | 30 | 2.11 | 70 |
| 24 | FLAIR | Siemens | SonataVision | McConnell_Brain_Imaging_Center | 2015-03-10T14:25:30.932490 | 1.5 | 3 | 0.079 | 9.4 | 180 |
| 24 | PD | Siemens | SonataVision | McConnell_Brain_Imaging_Center | 2015-03-10T14:21:17.189990 | 1.5 | 3 | 0.012 | 2.07 | 180 |
| 24 | T1w | Siemens | SonataVision | McConnell_Brain_Imaging_Center | 2015-03-10T14:14:10.512520 | 1.5 | 1 | 0.00399 | 3 | 8 |
| 24 | T2star | Siemens | SonataVision | McConnell_Brain_Imaging_Center | 2015-03-10T14:30:51.727490 | 1.5 | 3 | 0.032 | 0.65 | 20 |
| 24 | T2w | Siemens | SonataVision | McConnell_Brain_Imaging_Center | 2015-03-10T14:21:17.247480 | 1.5 | 3 | 0.083 | 2.07 | 180 |
| 24 | DWI | Siemens | SonataVision | McConnell_Brain_Imaging_Center | 2015-03-10T14:37:36.557520 | 1.5 | 2.2 | 0.078 | 7.8 | 90 |
| 24 | rsfMRI | Siemens | SonataVision | McConnell_Brain_Imaging_Center | 2015-03-10T14:06:14.670000 | 1.5 | 4.5 | 0.05 | 2.4 | 90 |
| 24 | rsfMRI | Siemens | SonataVision | McConnell_Brain_Imaging_Center | 2015-03-10T14:01:28.005010 | 1.5 | 4 | 0.05 | 2.73 | 90 |
| 24 | rsfMRI | Siemens | SonataVision | McConnell_Brain_Imaging_Center | 2015-03-10T14:10:27.570020 | 1.5 | 5 | 0.05 | 2.17 | 90 |
| 25 | FLAIR | Philips | Achieva | IRM_Quebec_-_Mailloux_-_3T | 2015-04-10T14:37:53.260000 | 3 | 3 | 0.125 | 9 | 90 |
| 25 | PD | Philips | Achieva | IRM_Quebec_-_Mailloux_-_3T | 2015-04-10T14:31:51.750000 | 3 | 3 | 0.0125 | 3 | 90 |
| 25 | T1w | Philips | Achieva | IRM_Quebec_-_Mailloux_-_3T | 2015-04-10T14:25:5.670000 | 3 | 1 | 0.003328 | 0.0073211 | 9 |
| 25 | T2star | Philips | Achieva | IRM_Quebec_-_Mailloux_-_3T | 2015-04-10T14:42:50.040000 | 3 | 3 | 0.02072 | 0.65 | 20 |
| 25 | T2w | Philips | Achieva | IRM_Quebec_-_Mailloux_-_3T | 2015-04-10T14:31:51.750000 | 3 | 3 | 0.1 | 3 | 90 |
| 25 | DWI | Philips | Achieva | IRM_Quebec_-_Mailloux_-_3T | 2015-04-10T14:47:22.870000 | 3 | 2 | 0.1024 | 10.1083 | 90 |
| 25 | rsfMRI | Philips | Achieva | IRM_Quebec_-_Mailloux_-_3T | 2015-04-10T14:55:45.310000 | 3 | 3.5 | 0.03 | 2.11 | 70 |
| 26 | FLAIR | Philips | Ingenia | CHUS_FLEURIMONT_Philips_3t | 2015-04-22T11:16:11.860000 | 3 | 3 | 0.125 | 9 | 90 |
| 26 | PD | Philips | Ingenia | CHUS_FLEURIMONT_Philips_3t | 2015-04-22T11:24:22.550000 | 3 | 3 | 0.0125 | 3 | 90 |
| 26 | T1w | Philips | Ingenia | CHUS_FLEURIMONT_Philips_3t | 2015-04-22T11:09:25.210000 | 3 | 1 | 0.0033 | 0.0072997 | 9 |
| 26 | T2star | Philips | Ingenia | CHUS_FLEURIMONT_Philips_3t | 2015-04-22T11:19:44.880000 | 3 | 3 | 0.020719 | 0.65 | 20 |
| 26 | T2w | Philips | Ingenia | CHUS_FLEURIMONT_Philips_3t | 2015-04-22T11:24:22.550000 | 3 | 3 | 0.1 | 3 | 90 |
| 26 | rsfMRI | Philips | Ingenia | CHUS_FLEURIMONT_Philips_3t | 2015-04-22T11:36:21.060000 | 3 | 3.5 | 0.03 | 2.11 | 70 |
| 27 | FLAIR | Siemens | TrioTim | Mc_Connell_Brain_Imaging_Centre | 2015-06-01T09:35:35.450000 | 3 | 3 | 0.123 | 9 | 165 |
| 27 | PD | Siemens | TrioTim | Mc_Connell_Brain_Imaging_Centre | 2015-06-01T09:40:2.095000 | 3 | 3 | 0.01 | 3 | 165 |
| 27 | T1w | Siemens | TrioTim | Mc_Connell_Brain_Imaging_Centre | 2015-06-01T09:28:51.495000 | 3 | 1 | 0.00298 | 2.3 | 9 |
| 27 | T2star | Siemens | TrioTim | Mc_Connell_Brain_Imaging_Centre | 2015-06-01T09:46:57.785000 | 3 | 3 | 0.02 | 0.65 | 20 |
| 27 | T2w | Siemens | TrioTim | Mc_Connell_Brain_Imaging_Centre | 2015-06-01T09:40:2.187500 | 3 | 3 | 0.091 | 3 | 165 |
| 27 | DWI | Siemens | TrioTim | Mc_Connell_Brain_Imaging_Centre | 2015-06-01T09:49:27.367500 | 3 | 2 | 0.096 | 9.4 | 90 |
| 27 | rsfMRI | Siemens | TrioTim | Mc_Connell_Brain_Imaging_Centre | 2015-06-01T09:56:32.790000 | 3 | 3.5 | 0.03 | 2.11 | 70 |
| 27 | rsfMRI | Siemens | OsiriX | Mc_Connell_Brain_Imaging_Centre | 2015-06-01T09:56:32.790000 | 3 | 3.5 | 0.03 | 2.11 | 70 |
| 28 | ASL | GE | DISCOVERY_MR750 | Concordia_University_Perform_Ctr | 2015-06-01T16:41:42.000000 | 3 | 4 | 0.010536 | 4.844 | 111 |
| 28 | FLAIR | GE | DISCOVERY_MR750 | Concordia_University_Perform_Ctr | 2015-06-01T16:10:39.000000 | 3 | 3 | 0.1435 | 9 | 125 |
| 28 | PD | GE | DISCOVERY_MR750 | Concordia_University_Perform_Ctr | 2015-06-01T16:05:7.000000 | 3 | 3 | 0.011224 | 3 | 125 |
| 28 | T1w | GE | DISCOVERY_MR750 | Concordia_University_Perform_Ctr | 2015-06-01T15:59:9.000000 | 3 | 1 | 0.002984 | 0.006872 | 11 |
| 28 | T2star | GE | DISCOVERY_MR750 | Concordia_University_Perform_Ctr | 2015-06-01T16:18:1.000000 | 3 | 3 | 0.02 | 0.65 | 20 |
| 28 | T2w | GE | DISCOVERY_MR750 | Concordia_University_Perform_Ctr | 2015-06-01T16:05:7.000000 | 3 | 3 | 0.089792 | 3 | 125 |
| 28 | DWI | GE | DISCOVERY_MR750 | Concordia_University_Perform_Ctr | 2015-06-01T16:35:46.000000 | 3 | 2.9 | 0.0838 | 9 | 90 |
| 28 | rsfMRI | GE | DISCOVERY_MR750 | Concordia_University_Perform_Ctr | 2015-06-01T16:24:4.000000 | 3 | 3.5 | 0.03 | 2.4 | 70 |
| 29 | ASL | GE | DISCOVERY_MR750 | b13b | 2015-10-20T10:56:16.000000 | 3 | 4 | 0.010536 | 6.639 | 111 |
| 29 | ASL | GE | DISCOVERY_MR750 | b13b | 2015-10-20T10:56:16.000000 | 3 | 4 | 0.010536 | 6.639 | 111 |
| 29 | ASL | GE | DISCOVERY_MR750 | b13b | 2015-10-20T10:56:16.000000 | 3 | 4 | 0.010536 | 6.639 | 111 |
| 29 | T1w | GE | DISCOVERY_MR750 | b13b | 2015-10-20T11:09:7.000000 | 3 | 1.3 | 0.089855 | 7 | 90 |
| 29 | T1w | GE | DISCOVERY_MR750 | b13b | 2015-10-20T09:13:3.000000 | 3 | 1 | 0.012794 | 0.65 | 90 |
| 29 | T1w | GE | DISCOVERY_MR750 | b13b | 2015-10-20T10:06:15.000000 | 3 | 0.8 | 0.015128 | 0.6 | 90 |
| 29 | T2w | GE | DISCOVERY_MR750 | b13b | 2015-10-20T10:43:14.000000 | 3 | 0.8 | 0.103719 | 3 | 90 |
| 29 | FLAIR | GE | DISCOVERY_MR750 | b13b | 2015-10-20T09:19:7.000000 | 3 | 3 | 0.136648 | 12.5 | 160 |
| 29 | PD | GE | DISCOVERY_MR750 | b13b | 2015-10-20T09:23:55.000000 | 3 | 3 | 0.01052 | 6.454 | 125 |
| 29 | T1w | GE | DISCOVERY_MR750 | b13b | 2015-10-20T10:49:45.000000 | 3 | 1 | 0.003036 | 0.00764 | 8 |
| 29 | T2w | GE | DISCOVERY_MR750 | b13b | 2015-10-20T09:23:55.000000 | 3 | 3 | 0.09468 | 6.454 | 125 |
| 29 | T1w | GE | DISCOVERY_MR750 | b13b | 2015-10-20T11:01:2.000000 | 3 | 1 | 0.003036 | 0.00764 | 8 |
| 29 | DWI | GE | DISCOVERY_MR750 | b13b | 2015-10-20T09:28:55.000000 | 3 | 2 | 0.0634 | 9 | 90 |
| 30 | T1w | Philips | Ingenia_CX | MRBSTTB17C | 2015-11-03T13:42:1.670000 | 3 | 0.6 | 0.005565 | 0.0118409 | 8 |
| 30 | T2w | Philips | Ingenia_CX | MRBSTTB17C | 2015-11-03T13:49:27.510000 | 3 | 0.6 | 0.361757 | 2.5 | 90 |
| 30 | T1w | Philips | Ingenia_CX | MRBSTTB17C | 2015-11-03T14:00:56.800000 | 3 | 0.6 | 0.005568 | 0.0118459 | 8 |
| 30 | ASL | Philips | Ingenia_CX | MRBSTTB17C | 2015-11-03T13:32:47.090000 | 3 | 5 | 0.018613 | 4.6023 | 90 |
| 30 | ASL | Philips | Ingenia_CX | MRBSTTB17C | 2015-11-03T13:41:38.970000 | 3 | 5 | 0.018613 | 4.6023 | 90 |
| 30 | ASL | Philips | Ingenia_CX | MRBSTTB17C | 2015-11-03T13:41:5.810000 | 3 | 5 | 0.016999 | 12 | 90 |
| 30 | SWI | Philips | Ingenia_CX | MRBSTTB17C | 2015-11-03T13:56:15.850000 | 3 | 2 |  | 0.031 | 17 |
| 30 | SWI | Philips | Ingenia_CX | MRBSTTB17C | 2015-11-03T13:56:15.850000 | 3 | 10 |  | 0.031 | 17 |
| 30 | SWI | Philips | Ingenia_CX | MRBSTTB17C | 2015-11-03T13:56:15.850000 | 3 | 2 |  | 0.031 | 17 |
| 30 | FLAIR | Philips | Ingenia_CX | MRBSTTB17C | 2015-11-03T13:05:29.830000 | 3 | 3 | 0.125 | 9 | 90 |
| 30 | PD | Philips | Ingenia_CX | MRBSTTB17C | 2015-11-03T13:00:22.520000 | 3 | 3 | 0.0125 | 3 | 90 |
| 30 | T1w | Philips | Ingenia_CX | MRBSTTB17C | 2015-11-03T12:53:19.930000 | 3 | 1 | 0.003298 | 0.0072756 | 9 |
| 30 | T2star | Philips | Ingenia_CX | MRBSTTB17C | 2015-11-03T13:10:3.740000 | 3 | 3 | 0.020721 | 0.65 | 20 |
| 30 | T2w | Philips | Ingenia_CX | MRBSTTB17C | 2015-11-03T13:00:22.520000 | 3 | 3 | 0.1 | 3 | 90 |
| 30 | DWI | Philips | Ingenia_CX | MRBSTTB17C | 2015-11-03T13:26:47.760000 | 3 | 2 | 0.079045 | 9.91052 | 90 |
| 30 | DWI | Philips | Ingenia | MRBSTTB09 | 2015-11-03T15:52:19.260000 | 3 | 2 | 0.110644 | 5.48428 | 90 |
| 30 | rsfMRI | Philips | Ingenia_CX | MRBSTTB17C | 2015-11-03T13:15:31.760000 | 3 | 3.5 | 0.030001 | 2.11 | 70 |
| 31 | FLAIR | Siemens | TrioTim | Hospital_Douglas | 2015-11-11T12:18:42.685000 | 3 | 3 | 0.123 | 9 | 165 |
| 31 | PD | Siemens | TrioTim | Hospital_Douglas | 2015-11-11T12:13:16.002500 | 3 | 3 | 0.01 | 3 | 165 |
| 31 | T1w | Siemens | TrioTim | Hospital_Douglas | 2015-11-11T12:07:40.490000 | 3 | 1 | 0.00298 | 2.3 | 9 |
| 31 | T2star | Siemens | TrioTim | Hospital_Douglas | 2015-11-11T12:24:11.160000 | 3 | 3 | 0.02 | 0.65 | 20 |
| 31 | T2w | Siemens | TrioTim | Hospital_Douglas | 2015-11-11T12:13:16.095000 | 3 | 3 | 0.091 | 3 | 165 |
| 31 | DWI | Siemens | TrioTim | Hospital_Douglas | 2015-11-11T12:26:24.357500 | 3 | 2 | 0.096 | 9.4 | 90 |
| 31 | rsfMRI | Siemens | TrioTim | Hospital_Douglas | 2015-11-11T12:33:29.035000 | 3 | 3.5 | 0.03 | 2.11 | 70 |
| 32 | T1w | Siemens | Skyra | Customer_Center | 2015-11-16T14:52:20.220000 | 3 | 0.83 | 0.00308 | 2.3 | 9 |
| 32 | T1w | Siemens | Skyra | Customer_Center | 2015-11-16T14:46:45.200000 | 3 | 1 | 0.00296 | 2.3 | 9 |
| 32 | ASL | Siemens | Skyra | Customer_Center | 2015-11-16T15:22:40.712500 | 3 | 8 | 0.012 | 2.5 | 90 |
| 32 | ASL | Siemens | Skyra | Customer_Center | 2015-11-16T15:22:40.712500 | 3 | 8 | 0.012 | 2.5 | 90 |
| 32 | ASL | Siemens | Skyra | Customer_Center | 2015-11-16T15:26:28.212500 | 3 | 8 | 0.012 | 2.5 | 90 |
| 32 | ASL | Siemens | Skyra | Customer_Center | 2015-11-16T15:26:28.212500 | 3 | 8 | 0.012 | 2.5 | 90 |
| 32 | SWI | Siemens | Skyra | Customer_Center | 2015-11-16T15:04:33.847500 | 3 | 12 | 0.02 | 0.027 | 15 |
| 32 | SWI | Siemens | Skyra | Customer_Center | 2015-11-16T15:04:33.847500 | 3 | 1.5 | 0.02 | 0.027 | 15 |
| 32 | FLAIR | Siemens | Skyra | Customer_Center | 2015-11-16T15:00:10.317500 | 3 | 3 | 0.13 | 9 | 165 |
| 32 | PD | Siemens | Skyra | Customer_Center | 2015-11-16T15:09:33.407500 | 3 | 3 | 0.091 | 3 | 165 |
| 32 | T2star | Siemens | Skyra | Customer_Center | 2015-11-16T15:21:2.067500 | 3 | 3 | 0.02 | 0.65 | 20 |
| 32 | T2w | Siemens | Skyra | Customer_Center | 2015-11-16T15:09:33.317500 | 3 | 3 | 0.01 | 3 | 165 |
| 32 | DWI | Siemens | Skyra | Customer_Center | 2015-11-16T15:28:24.240000 | 3 | 2 | 0.094 | 11.8 | 90 |
| 32 | rsfMRI | Siemens | Skyra | Customer_Center | 2015-11-16T16:40:41.540000 | 3 | 3.5 | 0.03 | 2.14 | 70 |
| 33 | T1w | Siemens | Prisma | Anlagenzentrum | 2015-11-17T09:24:9.295000 | 3 | 0.83 | 0.00308 | 2.3 | 9 |
| 33 | T1w | Siemens | Prisma | Anlagenzentrum | 2015-11-17T09:18:46.347500 | 3 | 1 | 0.00298 | 2.3 | 9 |
| 33 | ASL | Siemens | Prisma | Anlagenzentrum | 2015-11-17T09:49:11.630000 | 3 | 8 | 0.012 | 2.5 | 90 |
| 33 | ASL | Siemens | Prisma | Anlagenzentrum | 2015-11-17T09:52:59.130000 | 3 | 8 | 0.012 | 2.5 | 90 |
| 33 | ASL | Siemens | Prisma | Anlagenzentrum | 2015-11-17T09:52:59.130000 | 3 | 8 | 0.012 | 2.5 | 90 |
| 33 | PD | Siemens | Prisma | Anlagenzentrum | 2015-11-17T09:41:20.170000 | 3 | 3 | 0.011 | 3 | 165 |
| 33 | T2w | Siemens | Prisma | Anlagenzentrum | 2015-11-17T09:41:20.245000 | 3 | 3 | 0.098 | 3 | 165 |
| 33 | SWI | Siemens | Prisma | Anlagenzentrum | 2015-11-17T10:46:14.217500 | 3 | 1.5 | 0.02 | 0.027 | 15 |
| 33 | SWI | Siemens | Prisma | Anlagenzentrum | 2015-11-17T10:40:7.080000 | 3 | 1.5 | 0.02 | 0.027 | 15 |
| 33 | FLAIR | Siemens | Prisma | Anlagenzentrum | 2015-11-17T09:31:34.555000 | 3 | 3 | 0.13 | 9 | 165 |
| 33 | PD | Siemens | Prisma | Anlagenzentrum | 2015-11-17T09:36:1.162500 | 3 | 3 | 0.01 | 3 | 165 |
| 33 | T2star | Siemens | Prisma | Anlagenzentrum | 2015-11-17T09:47:30.090000 | 3 | 3 | 0.02 | 0.65 | 20 |
| 33 | T2w | Siemens | Prisma | Anlagenzentrum | 2015-11-17T09:36:1.252500 | 3 | 3 | 0.091 | 3 | 165 |
| 33 | DWI | Siemens | Prisma | Anlagenzentrum | 2015-11-17T09:54:7.602500 | 3 | 2 | 0.096 | 9.4 | 90 |
| 34 | T1w | Siemens | Prisma | Anlagenzentrum | 2015-11-17T11:15:45.060000 | 3 | 0.83 | 0.00308 | 2.3 | 9 |
| 34 | PD | Siemens | Prisma | Anlagenzentrum | 2015-11-17T11:23:6.045000 | 3 | 3 | 0.011 | 3 | 165 |
| 34 | T2w | Siemens | Prisma | Anlagenzentrum | 2015-11-17T11:23:6.120000 | 3 | 3 | 0.098 | 3 | 165 |
| 35 | FLAIR | Philips | Intera | UBC | 2016-10-03T17:00:34.370000 | 3 | 3 | 0.125 | 9 | 90 |
| 35 | PD | Philips | Intera | UBC | 2016-10-03T16:54:32.640000 | 3 | 3 | 0.0125 | 3 | 90 |
| 35 | T1w | Philips | Intera | UBC | 2016-10-03T16:47:37.620000 | 3 | 1 | 0.00328 | 0.0072515 | 9 |
| 35 | T2star | Philips | Intera | UBC | 2016-10-03T17:05:32.030000 | 3 | 3 | 0.020714 | 0.65 | 20 |
| 35 | T2w | Philips | Intera | UBC | 2016-10-03T16:54:32.640000 | 3 | 3 | 0.1 | 3 | 90 |
| 35 | DWI | Philips | Intera | UBC | 2016-10-03T17:11:4.420000 | 3 | 2 | 0.100847 | 9.97048 | 90 |
| 35 | rsfMRI | Philips | Intera | UBC | 2016-10-03T17:17:58.810000 | 3 | 3.5 | 0.030001 | 2.11 | 70 |
| 36 | FLAIR | Siemens | TrioTim | IUGM | 2016-10-25T10:15:54.690000 | 3 | 3 | 0.123 | 9 | 165 |
| 36 | T1w | Siemens | TrioTim | IUGM | 2016-10-25T10:08:55.950000 | 3 | 1 | 0.00298 | 2.3 | 9 |
| 36 | T2star | Siemens | TrioTim | IUGM | 2016-10-25T10:21:26.890000 | 3 | 3 | 0.02 | 0.65 | 20 |
| 36 | FLAIR | Siemens | TrioTim | IUGM | 2016-10-25T12:18:20.687500 | 3 | 3 | 0.123 | 9 | 165 |
| 36 | T1w | Siemens | TrioTim | IUGM | 2016-10-25T12:11:53.652500 | 3 | 1 | 0.00298 | 2.3 | 9 |
| 36 | T2star | Siemens | TrioTim | IUGM | 2016-10-25T12:23:49.147500 | 3 | 3 | 0.02 | 0.65 | 20 |
| 36 | FLAIR | Siemens | TrioTim | IUGM | 2016-10-25T13:02:35.902500 | 3 | 3 | 0.123 | 9 | 165 |
| 36 | T1w | Siemens | TrioTim | IUGM | 2016-10-25T12:55:51.440000 | 3 | 1 | 0.00298 | 2.3 | 9 |
| 36 | T2star | Siemens | TrioTim | IUGM | 2016-10-25T13:08:4.165000 | 3 | 3 | 0.02 | 0.65 | 20 |
| 36 | FLAIR | Siemens | TrioTim | IUGM | 2016-10-25T15:37:49.305000 | 3 | 3 | 0.123 | 9 | 165 |
| 36 | T1w | Siemens | TrioTim | IUGM | 2016-10-25T15:31:27.420000 | 3 | 1 | 0.00298 | 2.3 | 9 |
| 36 | T2star | Siemens | TrioTim | IUGM | 2016-10-25T15:43:17.157500 | 3 | 3 | 0.02 | 0.65 | 20 |
| 36 | FLAIR | Siemens | TrioTim | IUGM | 2016-10-25T16:22:16.262500 | 3 | 3 | 0.123 | 9 | 165 |
| 36 | T1w | Siemens | TrioTim | IUGM | 2016-10-25T16:15:57.415000 | 3 | 1 | 0.00298 | 2.3 | 9 |
| 36 | T2star | Siemens | TrioTim | IUGM | 2016-10-25T16:27:45.165000 | 3 | 3 | 0.02 | 0.65 | 20 |
| 36 | DWI | Siemens | TrioTim | IUGM | 2016-10-25T10:23:57.397500 | 3 | 2 | 0.096 | 9.4 | 90 |
| 36 | DWI | Siemens | TrioTim | IUGM | 2016-10-25T12:26:14.360000 | 3 | 2 | 0.096 | 9.4 | 90 |
| 36 | DWI | Siemens | TrioTim | IUGM | 2016-10-25T13:10:24.370000 | 3 | 2 | 0.096 | 9.4 | 90 |
| 36 | DWI | Siemens | TrioTim | IUGM | 2016-10-25T15:45:38.365000 | 3 | 2 | 0.096 | 9.4 | 90 |
| 36 | DWI | Siemens | TrioTim | IUGM | 2016-10-25T16:30:6.367500 | 3 | 2 | 0.096 | 9.4 | 90 |
| 36 | rsfMRI | Siemens | TrioTim | IUGM | 2016-10-25T10:31:19.865000 | 3 | 3.5 | 0.03 | 2.11 | 70 |
| 36 | rsfMRI | Siemens | TrioTim | IUGM | 2016-10-25T12:33:21.717500 | 3 | 3.5 | 0.03 | 2.11 | 70 |
| 36 | rsfMRI | Siemens | TrioTim | IUGM | 2016-10-25T13:17:19.815000 | 3 | 3.5 | 0.03 | 2.11 | 70 |
| 36 | rsfMRI | Siemens | TrioTim | IUGM | 2016-10-25T15:53:56.842500 | 3 | 3.5 | 0.03 | 2.11 | 70 |
| 36 | rsfMRI | Siemens | TrioTim | IUGM | 2016-10-25T16:37:12.995000 | 3 | 3.5 | 0.03 | 2.11 | 70 |
| 37 | FLAIR | Philips | Ingenia | CHUS_FLEURIMONT_Philips_3t | 2016-11-03T09:44:57.880000 | 3 | 3 | 0.125 | 9 | 90 |
| 37 | PD | Philips | Ingenia | CHUS_FLEURIMONT_Philips_3t | 2016-11-03T09:39:31.610000 | 3 | 3 | 0.0125 | 3 | 90 |
| 37 | T1w | Philips | Ingenia | CHUS_FLEURIMONT_Philips_3t | 2016-11-03T09:32:58.520000 | 3 | 1 | 0.003328 | 0.0073444 | 9 |
| 37 | T2star | Philips | Ingenia | CHUS_FLEURIMONT_Philips_3t | 2016-11-03T09:48:29.570000 | 3 | 3 | 0.020719 | 0.65 | 20 |
| 37 | T2w | Philips | Ingenia | CHUS_FLEURIMONT_Philips_3t | 2016-11-03T09:39:31.610000 | 3 | 3 | 0.1 | 3 | 90 |
| 37 | DWI | Philips | Ingenia | CHUS_FLEURIMONT_Philips_3t | 2016-11-03T10:04:30.320000 | 3 | 2 | 0.1075 | 10.0087 | 90 |
| 37 | rsfMRI | Philips | Ingenia | CHUS_FLEURIMONT_Philips_3t | 2016-11-03T09:53:17.230000 | 3 | 3.5 | 0.03 | 2.11 | 70 |
| 38 | FLAIR | Philips | Achieva | IRM_Quebec_-_Mailloux_-_3T | 2016-11-16T16:25:34.290000 | 3 | 3 | 0.125 | 9 | 90 |
| 38 | PD | Philips | Achieva | IRM_Quebec_-_Mailloux_-_3T | 2016-11-16T16:30:43.100000 | 3 | 3 | 0.0125 | 3 | 90 |
| 38 | T1w | Philips | Achieva | IRM_Quebec_-_Mailloux_-_3T | 2016-11-16T16:18:40.460000 | 3 | 1 | 0.003327 | 0.0073306 | 9 |
| 38 | T2star | Philips | Achieva | IRM_Quebec_-_Mailloux_-_3T | 2016-11-16T16:36:55.810000 | 3 | 3 | 0.020721 | 0.65 | 20 |
| 38 | T2w | Philips | Achieva | IRM_Quebec_-_Mailloux_-_3T | 2016-11-16T16:30:43.100000 | 3 | 3 | 0.1 | 3 | 90 |
| 38 | FLAIR | Philips | Achieva | IRM_Quebec_-_Mailloux_-_3T | 2016-11-16T16:41:29.450000 | 3 | 3 | 0.125 | 9 | 90 |
| 38 | PD | Philips | Achieva | IRM_Quebec_-_Mailloux_-_3T | 2016-11-16T16:45:55.250000 | 3 | 3 | 0.0125 | 3 | 90 |
| 38 | T2w | Philips | Achieva | IRM_Quebec_-_Mailloux_-_3T | 2016-11-16T16:45:55.250000 | 3 | 3 | 0.1 | 3 | 90 |
| 38 | DWI | Philips | Achieva | IRM_Quebec_-_Mailloux_-_3T | 2016-11-16T16:51:39.920000 | 3 | 2 | 0.1024 | 10.1083 | 90 |
| 38 | rsfMRI | Philips | Achieva | IRM_Quebec_-_Mailloux_-_3T | 2016-11-16T17:00:36.040000 | 3 | 3.5 | 0.030001 | 2.11 | 70 |
| 39 | FLAIR | Siemens | TrioTim | Mc_Connell_Brain_Imaging_Centre | 2016-11-22T10:19:58.950000 | 3 | 3 | 0.123 | 9 | 165 |
| 39 | PD | Siemens | TrioTim | Mc_Connell_Brain_Imaging_Centre | 2016-11-22T10:23:56.245000 | 3 | 3 | 0.01 | 3 | 165 |
| 39 | T1w | Siemens | TrioTim | Mc_Connell_Brain_Imaging_Centre | 2016-11-22T10:13:14.640000 | 3 | 1 | 0.00298 | 2.3 | 9 |
| 39 | T2star | Siemens | TrioTim | Mc_Connell_Brain_Imaging_Centre | 2016-11-22T10:30:45.207500 | 3 | 3 | 0.02 | 0.65 | 20 |
| 39 | T2w | Siemens | TrioTim | Mc_Connell_Brain_Imaging_Centre | 2016-11-22T10:23:56.337500 | 3 | 3 | 0.091 | 3 | 165 |
| 39 | DWI | Siemens | TrioTim | Mc_Connell_Brain_Imaging_Centre | 2016-11-22T10:33:8.192500 | 3 | 2 | 0.096 | 9.4 | 90 |
| 39 | rsfMRI | Siemens | TrioTim | Mc_Connell_Brain_Imaging_Centre | 2016-11-22T10:40:5.677500 | 3 | 3.5 | 0.03 | 2.11 | 70 |
| 40 | FLAIR | Siemens | TrioTim | Hospital_Douglas | 2016-11-22T12:41:23.360000 | 3 | 3 | 0.123 | 9 | 165 |
| 40 | PD | Siemens | TrioTim | Hospital_Douglas | 2016-11-22T12:35:54.365000 | 3 | 3 | 0.01 | 3 | 165 |
| 40 | T1w | Siemens | TrioTim | Hospital_Douglas | 2016-11-22T12:30:2.815000 | 3 | 1 | 0.00298 | 2.3 | 9 |
| 40 | T2star | Siemens | TrioTim | Hospital_Douglas | 2016-11-22T12:46:54.182500 | 3 | 3 | 0.02 | 0.65 | 20 |
| 40 | T2w | Siemens | TrioTim | Hospital_Douglas | 2016-11-22T12:35:54.457500 | 3 | 3 | 0.091 | 3 | 165 |
| 40 | DWI | Siemens | TrioTim | Hospital_Douglas | 2016-11-22T12:49:8.675000 | 3 | 2 | 0.096 | 9.4 | 90 |
| 40 | rsfMRI | Siemens | TrioTim | Hospital_Douglas | 2016-11-22T12:55:58.735000 | 3 | 3.5 | 0.03 | 2.11 | 70 |
| 41 | FLAIR | Philips | Achieva | CHUM_Campus_Notre-Dame | 2016-11-22T15:55:55.750000 | 3 | 3 | 0.125 | 9 | 90 |
| 41 | PD | Philips | Achieva | CHUM_Campus_Notre-Dame | 2016-11-22T15:49:58.790000 | 3 | 3 | 0.0125 | 3 | 90 |
| 41 | T1w | Philips | Achieva | CHUM_Campus_Notre-Dame | 2016-11-22T15:42:11.450000 | 3 | 1 | 0.003295 | 0.007267 | 9 |
| 41 | T2star | Philips | Achieva | CHUM_Campus_Notre-Dame | 2016-11-22T16:00:53.010000 | 3 | 3 | 0.020714 | 0.65 | 20 |
| 41 | T2w | Philips | Achieva | CHUM_Campus_Notre-Dame | 2016-11-22T15:49:58.790000 | 3 | 3 | 0.1 | 3 | 90 |
| 41 | DWI | Philips | Achieva | CHUM_Campus_Notre-Dame | 2016-11-22T16:06:30.420000 | 3 | 2 | 0.100847 | 9.96914 | 90 |
| 41 | rsfMRI | Philips | Achieva | CHUM_Campus_Notre-Dame | 2016-11-22T16:13:3.930000 | 3 | 3.5 | 0.03 | 2.11 | 70 |
| 42 | FLAIR | GE | DISCOVERY_MR750 | FOOTHILLS_Hospital | 2016-11-28T12:20:34.000000 | 3 | 3 | 0.1435 | 9 | 125 |
| 42 | PD | GE | DISCOVERY_MR750 | FOOTHILLS_Hospital | 2016-11-28T12:17:33.000000 | 3 | 3 | 0.011224 | 3 | 125 |
| 42 | T1w | GE | DISCOVERY_MR750 | FOOTHILLS_Hospital | 2016-11-28T12:11:9.000000 | 3 | 1 | 0.003084 | 0.007472 | 11 |
| 42 | T2star | GE | DISCOVERY_MR750 | FOOTHILLS_Hospital | 2016-11-28T12:28:43.000000 | 3 | 3 | 0.02 | 0.65 | 20 |
| 42 | T2w | GE | DISCOVERY_MR750 | FOOTHILLS_Hospital | 2016-11-28T12:17:33.000000 | 3 | 3 | 0.089792 | 3 | 125 |
| 42 | FLAIR | GE | DISCOVERY_MR750 | FOOTHILLS_Hospital | 2016-11-28T13:11:15.000000 | 3 | 3 | 0.14378 | 9 | 125 |
| 42 | PD | GE | DISCOVERY_MR750 | FOOTHILLS_Hospital | 2016-11-28T13:08:12.000000 | 3 | 3 | 0.011224 | 3 | 125 |
| 42 | T1w | GE | DISCOVERY_MR750 | FOOTHILLS_Hospital | 2016-11-28T13:01:45.000000 | 3 | 1 | 0.003052 | 0.007384 | 11 |
| 42 | T2star | GE | DISCOVERY_MR750 | FOOTHILLS_Hospital | 2016-11-28T13:17:36.000000 | 3 | 3 | 0.0149 | 0.65 | 20 |
| 42 | T2w | GE | DISCOVERY_MR750 | FOOTHILLS_Hospital | 2016-11-28T13:08:12.000000 | 3 | 3 | 0.089792 | 3 | 125 |
| 42 | DWI | GE | DISCOVERY_MR750 | FOOTHILLS_Hospital | 2016-11-28T12:31:34.000000 | 3 | 2 | 0.0856 | 9 | 90 |
| 42 | DWI | GE | DISCOVERY_MR750 | FOOTHILLS_Hospital | 2016-11-28T13:20:18.000000 | 3 | 2 | 0.084 | 9 | 90 |
| 42 | rsfMRI | GE | DISCOVERY_MR750 | FOOTHILLS_Hospital | 2016-11-28T12:38:6.000000 | 3 | 3.5 | 0.03 | 2.5 | 70 |
| 43 | FLAIR | Siemens | Prisma | Peter_S._Allen_MR_Research_Centre | 2016-11-29T14:56:46.477500 | 3 | 3 | 0.12 | 9 | 165 |
| 43 | PD | Siemens | Prisma | Peter_S._Allen_MR_Research_Centre | 2016-11-29T14:53:26.742500 | 3 | 3 | 0.01 | 3 | 165 |
| 43 | T1w | Siemens | Prisma | Peter_S._Allen_MR_Research_Centre | 2016-11-29T14:46:58.585000 | 3 | 1 | 0.00298 | 2.3 | 9 |
| 43 | T2star | Siemens | Prisma | Peter_S._Allen_MR_Research_Centre | 2016-11-29T15:00:54.955000 | 3 | 3 | 0.02 | 0.65 | 20 |
| 43 | T2w | Siemens | Prisma | Peter_S._Allen_MR_Research_Centre | 2016-11-29T14:53:26.835000 | 3 | 3 | 0.093 | 3 | 165 |
| 43 | FLAIR | Siemens | Prisma | Peter_S._Allen_MR_Research_Centre | 2016-11-29T15:25:42.455000 | 3 | 3 | 0.12 | 9 | 165 |
| 43 | DWI | Siemens | Prisma | Peter_S._Allen_MR_Research_Centre | 2016-11-29T15:14:48.525000 | 3 | 2 | 0.064 | 9.4 | 90 |
| 43 | DWI | Siemens | Prisma | Peter_S._Allen_MR_Research_Centre | 2016-11-29T15:21:40.712500 | 3 | 2 | 0.064 | 6.9 | 90 |
| 43 | rsfMRI | Siemens | Prisma | Peter_S._Allen_MR_Research_Centre | 2016-11-29T15:03:11.780000 | 3 | 3.5 | 0.03 | 2.13 | 70 |
| 44 | FLAIR | Siemens | TrioTim | Mc_Connell_Brain_Imaging_Centre | 2017-01-13T09:56:23.305000 | 3 | 3 | 0.123 | 9 | 165 |
| 44 | PD | Siemens | TrioTim | Mc_Connell_Brain_Imaging_Centre | 2017-01-13T10:00:20.292500 | 3 | 3 | 0.01 | 3 | 165 |
| 44 | T1w | Siemens | TrioTim | Mc_Connell_Brain_Imaging_Centre | 2017-01-13T09:37:39.282500 | 3 | 1 | 0.00298 | 2.3 | 9 |
| 44 | T2star | Siemens | TrioTim | Mc_Connell_Brain_Imaging_Centre | 2017-01-13T10:07:11.592500 | 3 | 3 | 0.02 | 0.65 | 20 |
| 44 | T2w | Siemens | TrioTim | Mc_Connell_Brain_Imaging_Centre | 2017-01-13T10:00:20.382500 | 3 | 3 | 0.091 | 3 | 165 |
| 44 | FLAIR | Siemens | TrioTim | Mc_Connell_Brain_Imaging_Centre | 2017-01-13T10:34:11.997500 | 3 | 3 | 0.123 | 9 | 165 |
| 44 | PD | Siemens | TrioTim | Mc_Connell_Brain_Imaging_Centre | 2017-01-13T10:38:9.452500 | 3 | 3 | 0.01 | 3 | 165 |
| 44 | T1w | Siemens | TrioTim | Mc_Connell_Brain_Imaging_Centre | 2017-01-13T10:15:42.185000 | 3 | 1 | 0.00298 | 2.3 | 9 |
| 44 | T2star | Siemens | TrioTim | Mc_Connell_Brain_Imaging_Centre | 2017-01-13T10:44:58.340000 | 3 | 3 | 0.02 | 0.65 | 20 |
| 44 | T2w | Siemens | TrioTim | Mc_Connell_Brain_Imaging_Centre | 2017-01-13T10:38:9.542500 | 3 | 3 | 0.091 | 3 | 165 |
| 44 | FLAIR | Siemens | TrioTim | Mc_Connell_Brain_Imaging_Centre | 2017-01-13T11:23:0.280000 | 3 | 3 | 0.123 | 9 | 165 |
| 44 | PD | Siemens | TrioTim | Mc_Connell_Brain_Imaging_Centre | 2017-01-13T11:26:57.222500 | 3 | 3 | 0.01 | 3 | 165 |
| 44 | T1w | Siemens | TrioTim | Mc_Connell_Brain_Imaging_Centre | 2017-01-13T11:03:26.945000 | 3 | 1 | 0.00298 | 2.3 | 9 |
| 44 | T2star | Siemens | TrioTim | Mc_Connell_Brain_Imaging_Centre | 2017-01-13T11:33:48.650000 | 3 | 3 | 0.02 | 0.65 | 20 |
| 44 | T2w | Siemens | TrioTim | Mc_Connell_Brain_Imaging_Centre | 2017-01-13T11:26:57.315000 | 3 | 3 | 0.091 | 3 | 165 |
| 44 | DWI | Siemens | TrioTim | Mc_Connell_Brain_Imaging_Centre | 2017-01-13T10:09:25.372500 | 3 | 2 | 0.096 | 9.4 | 90 |
| 44 | DWI | Siemens | TrioTim | Mc_Connell_Brain_Imaging_Centre | 2017-01-13T10:47:11.375000 | 3 | 2 | 0.096 | 9.4 | 90 |
| 44 | DWI | Siemens | TrioTim | Mc_Connell_Brain_Imaging_Centre | 2017-01-13T11:36:2.375000 | 3 | 2 | 0.096 | 9.4 | 90 |
| 44 | rsfMRI | Siemens | TrioTim | Mc_Connell_Brain_Imaging_Centre | 2017-01-13T09:43:56.117500 | 3 | 3.5 | 0.03 | 2.11 | 70 |
| 44 | rsfMRI | Siemens | TrioTim | Mc_Connell_Brain_Imaging_Centre | 2017-01-13T10:21:42.027500 | 3 | 3.5 | 0.03 | 2.11 | 70 |
| 44 | rsfMRI | Siemens | TrioTim | Mc_Connell_Brain_Imaging_Centre | 2017-01-13T11:10:14.210000 | 3 | 3.5 | 0.03 | 2.11 | 70 |
| 45 | FLAIR | Siemens | Prisma_fit | IUGM | 2017-01-19T10:48:51.025000 | 3 | 3 | 0.12 | 9 | 165 |
| 45 | T1w | Siemens | Prisma_fit | IUGM | 2017-01-19T10:42:45.630000 | 3 | 1 | 0.00296 | 2.3 | 9 |
| 45 | T2star | Siemens | Prisma_fit | IUGM | 2017-01-19T10:54:19.615000 | 3 | 3 | 0.02 | 0.65 | 20 |
| 45 | FLAIR | Siemens | Prisma_fit | IUGM | 2017-01-19T11:28:16.125000 | 3 | 3 | 0.12 | 9 | 165 |
| 45 | T1w | Siemens | Prisma_fit | IUGM | 2017-01-19T11:22:36.362500 | 3 | 1 | 0.00296 | 2.3 | 9 |
| 45 | T2star | Siemens | Prisma_fit | IUGM | 2017-01-19T11:33:41.045000 | 3 | 3 | 0.02 | 0.65 | 20 |
| 45 | DWI | Siemens | Prisma_fit | IUGM | 2017-01-19T10:57:25.142500 | 3 | 2 | 0.096 | 9.4 | 90 |
| 45 | DWI | Siemens | Prisma_fit | IUGM | 2017-01-19T11:36:9.725000 | 3 | 2 | 0.096 | 9.4 | 90 |
| 45 | rsfMRI | Siemens | Prisma_fit | IUGM | 2017-01-19T11:03:13.117500 | 3 | 3.5 | 0.03 | 2.13 | 70 |
| 45 | rsfMRI | Siemens | Prisma_fit | IUGM | 2017-01-19T11:41:44.582500 | 3 | 3.5 | 0.03 | 2.13 | 70 |
| 46 | T2star | Siemens | Prisma_fit | IUGM | 2017-01-19T15:54:5.070000 | 3 | 3 | 0.02 | 0.65 | 20 |
| 46 | FLAIR | Siemens | Prisma_fit | IUGM | 2017-01-19T14:24:34.990000 | 3 | 3 | 0.12 | 9 | 165 |
| 46 | T1w | Siemens | Prisma_fit | IUGM | 2017-01-19T14:17:54.240000 | 3 | 1 | 0.00296 | 2.3 | 9 |
| 46 | T2star | Siemens | Prisma_fit | IUGM | 2017-01-19T14:30:0.030000 | 3 | 3 | 0.02 | 0.65 | 20 |
| 46 | FLAIR | Siemens | Prisma_fit | IUGM | 2017-01-19T15:05:4.775000 | 3 | 3 | 0.12 | 9 | 165 |
| 46 | T1w | Siemens | Prisma_fit | IUGM | 2017-01-19T14:59:19.257500 | 3 | 1 | 0.00296 | 2.3 | 9 |
| 46 | T2star | Siemens | Prisma_fit | IUGM | 2017-01-19T15:10:30.037500 | 3 | 3 | 0.02 | 0.65 | 20 |
| 46 | FLAIR | Siemens | Prisma_fit | IUGM | 2017-01-19T15:49:59.512500 | 3 | 3 | 0.12 | 9 | 165 |
| 46 | PD | Siemens | Prisma_fit | IUGM | 2017-01-19T15:46:38.132500 | 3 | 3 | 0.01 | 3 | 165 |
| 46 | T1w | Siemens | Prisma_fit | IUGM | 2017-01-19T15:40:25.007500 | 3 | 1 | 0.00298 | 2.3 | 9 |
| 46 | T2star | Siemens | Prisma_fit | IUGM | 2017-01-19T15:54:5.070000 | 3 | 3 | 0.02 | 0.65 | 20 |
| 46 | T2w | Siemens | Prisma_fit | IUGM | 2017-01-19T15:46:38.225000 | 3 | 3 | 0.093 | 3 | 165 |
| 46 | DWI | Siemens | Prisma_fit | IUGM | 2017-01-19T14:32:26.250000 | 3 | 2 | 0.096 | 9.4 | 90 |
| 46 | DWI | Siemens | Prisma_fit | IUGM | 2017-01-19T15:12:58.712500 | 3 | 2 | 0.096 | 9.4 | 90 |
| 46 | DWI | Siemens | Prisma_fit | IUGM | 2017-01-19T16:09:5.987500 | 3 | 2 | 0.064 | 9.4 | 90 |
| 46 | DWI | Siemens | Prisma_fit | IUGM | 2017-01-19T16:15:52.307500 | 3 | 2 | 0.064 | 6.9 | 90 |
| 46 | rsfMRI | Siemens | Prisma_fit | IUGM | 2017-01-19T14:38:16.037500 | 3 | 3.5 | 0.03 | 2.13 | 70 |
| 46 | rsfMRI | Siemens | Prisma_fit | IUGM | 2017-01-19T15:18:43.177500 | 3 | 3.5 | 0.03 | 2.13 | 70 |
| 46 | rsfMRI | Siemens | Prisma_fit | IUGM | 2017-01-19T15:56:33.605000 | 3 | 3.5 | 0.03 | 2.13 | 70 |
| 47 | T2star | Siemens | Prisma | Sunnybrook_Research_Institute | 2017-02-22T14:40:36.970000 | 3 | 3 | 0.02 | 0.65 | 20 |
| 47 | FLAIR | Siemens | Prisma | Sunnybrook_Research_Institute | 2017-02-22T14:36:28.445000 | 3 | 3 | 0.12 | 9 | 165 |
| 47 | PD | Siemens | Prisma | Sunnybrook_Research_Institute | 2017-02-22T14:33:3.905000 | 3 | 3 | 0.01 | 3 | 165 |
| 47 | T1w | Siemens | Prisma | Sunnybrook_Research_Institute | 2017-02-22T14:26:35.680000 | 3 | 1 | 0.00298 | 2.3 | 9 |
| 47 | T2star | Siemens | Prisma | Sunnybrook_Research_Institute | 2017-02-22T14:40:36.970000 | 3 | 3 | 0.02 | 0.65 | 20 |
| 47 | T2w | Siemens | Prisma | Sunnybrook_Research_Institute | 2017-02-22T14:33:3.997500 | 3 | 3 | 0.093 | 3 | 165 |
| 47 | DWI | Siemens | Prisma | Sunnybrook_Research_Institute | 2017-02-22T14:54:15.530000 | 3 | 2 | 0.064 | 6.9 | 90 |
| 47 | rsfMRI | Siemens | Prisma | Sunnybrook_Research_Institute | 2017-02-22T14:43:5.685000 | 3 | 3.5 | 0.03 | 2.13 | 70 |
| 48 | T1w | Siemens | Allegra | Cuban Neuroscience Center | 2017-03-14T16:01:25.692500 | 3 | 1 | 0.00529 | 0.0096 | 15 |
| 48 | FLAIR | Siemens | Allegra | Cuban Neuroscience Center | 2017-03-14T15:39:25.092500 | 3 | 3 | 0.122 | 9 | 120 |
| 48 | PD | Siemens | Allegra | Cuban Neuroscience Center | 2017-03-14T15:21:52.587490 | 3 | 3 | 0.012 | 3 | 141 |
| 48 | T1w | Siemens | Allegra | Cuban Neuroscience Center | 2017-03-14T15:16:23.959980 | 3 | 1 | 0.00286 | 2.3 | 9 |
| 48 | T2star | Siemens | Allegra | Cuban Neuroscience Center | 2017-03-14T15:28:45.597480 | 3 | 3 | 0.02 | 0.65 | 20 |
| 48 | T2w | Siemens | Allegra | Cuban Neuroscience Center | 2017-03-14T15:21:52.672500 | 3 | 3 | 0.096 | 3 | 141 |
| 48 | T1w | Siemens | Allegra | Cuban Neuroscience Center | 2017-03-14T15:55:59.957510 | 3 | 1 | 0.00286 | 2.3 | 9 |
| 48 | rsfMRI | Siemens | Allegra | Cuban Neuroscience Center | 2017-03-14T15:45:25.414990 | 3 | 3.5 | 0.023 | 2.1 | 70 |
| 49 | T1w | Siemens | Allegra | Cuban Neuroscience Center | 2017-03-16T09:19:46.984990 | 3 | 1 | 0.00283 | 2.3 | 9 |
| 50 | T2star | Siemens | Prisma_fit | Robarts_Research_Institute | 2017-04-03T13:36:20.970000 | 3 | 3 | 0.02 | 0.65 | 20 |
| 50 | FLAIR | Siemens | Prisma_fit | Robarts_Research_Institute | 2017-04-03T13:30:23.447500 | 3 | 3 | 0.12 | 9 | 165 |
| 50 | PD | Siemens | Prisma_fit | Robarts_Research_Institute | 2017-04-03T13:26:59.752500 | 3 | 3 | 0.01 | 3 | 165 |
| 50 | T1w | Siemens | Prisma_fit | Robarts_Research_Institute | 2017-04-03T13:21:22.742500 | 3 | 1 | 0.00298 | 2.3 | 9 |
| 50 | T2star | Siemens | Prisma_fit | Robarts_Research_Institute | 2017-04-03T13:36:20.970000 | 3 | 3 | 0.02 | 0.65 | 20 |
| 50 | T2w | Siemens | Prisma_fit | Robarts_Research_Institute | 2017-04-03T13:26:59.845000 | 3 | 3 | 0.093 | 3 | 165 |
| 50 | DWI | Siemens | Prisma_fit | Robarts_Research_Institute | 2017-04-03T13:48:13.327500 | 3 | 2 | 0.064 | 6.9 | 90 |
| 50 | rsfMRI | Siemens | Prisma_fit | Robarts_Research_Institute | 2017-04-03T13:38:37.392500 | 3 | 3.5 | 0.03 | 2.13 | 70 |
| 51 | FLAIR | Siemens | Prisma_fit | Mc_Connell_Brain_Imaging_Centre | 2017-06-06T09:40:25.397500 | 3 | 3 | 0.12 | 9 | 165 |
| 51 | T1w | Siemens | Prisma_fit | Mc_Connell_Brain_Imaging_Centre | 2017-06-06T09:34:34.132500 | 3 | 1 | 0.00296 | 2.3 | 9 |
| 51 | T2star | Siemens | Prisma_fit | Mc_Connell_Brain_Imaging_Centre | 2017-06-06T09:45:59.777500 | 3 | 3 | 0.02 | 0.65 | 20 |
| 51 | FLAIR | Siemens | Prisma_fit | Mc_Connell_Brain_Imaging_Centre | 2017-06-06T10:18:31.155000 | 3 | 3 | 0.12 | 9 | 165 |
| 51 | T1w | Siemens | Prisma_fit | Mc_Connell_Brain_Imaging_Centre | 2017-06-06T10:12:41.970000 | 3 | 1 | 0.00296 | 2.3 | 9 |
| 51 | T2star | Siemens | Prisma_fit | Mc_Connell_Brain_Imaging_Centre | 2017-06-06T10:24:1.070000 | 3 | 3 | 0.02 | 0.65 | 20 |
| 51 | DWI | Siemens | Prisma_fit | Mc_Connell_Brain_Imaging_Centre | 2017-06-06T09:48:35.180000 | 3 | 2 | 0.096 | 9.4 | 90 |
| 51 | DWI | Siemens | Prisma_fit | Mc_Connell_Brain_Imaging_Centre | 2017-06-06T10:26:31.785000 | 3 | 2 | 0.096 | 9.4 | 90 |
| 51 | rsfMRI | Siemens | Prisma_fit | Mc_Connell_Brain_Imaging_Centre | 2017-06-06T09:55:11.655000 | 3 | 3.5 | 0.03 | 2.13 | 70 |
| 51 | rsfMRI | Siemens | Prisma_fit | Mc_Connell_Brain_Imaging_Centre | 2017-06-06T10:32:2.475000 | 3 | 3.5 | 0.03 | 2.13 | 70 |
| 52 | FLAIR | Siemens | Skyra | 3T_ROYAL_UNIVERSITY_HOSP | 2017-06-22T16:40:06.217500 | 3 | 3 | 125 | 9000 | 165 |
| 52 | PD | Siemens | Skyra | 3T_ROYAL_UNIVERSITY_HOSP | 2017-06-22T16:48:16.935000 | 3 | 3 | 10 | 3000 | 165 |
| 52 | T1w | Siemens | Skyra | 3T_ROYAL_UNIVERSITY_HOSP | 2017-06-22T16:32:23.060000 | 3 | 1 | 0.00298 | 2.3 | 9 |
| 52 | T2star | Siemens | Skyra | 3T_ROYAL_UNIVERSITY_HOSP | 2017-06-22T16:55:05.507500 | 3 | 3 | 0.02 | 0.65 | 20 |
| 52 | T2w | Siemens | Skyra | 3T_ROYAL_UNIVERSITY_HOSP | 2017-06-22T16:48:17.027500 | 3 | 3 | 0.093 | 3 | 165 |
| 52 | FLAIR | Siemens | Skyra | 3T_ROYAL_UNIVERSITY_HOSP | 2017-06-22T16:44:17.105000 | 3 | 3 | 125 | 9000 | 165 |
| 52 | DWI | Siemens | Skyra | 3T_ROYAL_UNIVERSITY_HOSP | 2017-06-22T17:12:18.151 | 3 | 2 | 0.101 | 13 | 90 |
| 52 | rsfMRI | Siemens | Skyra | 3T_ROYAL_UNIVERSITY_HOSP | 2017-06-22T16:56:59.055000 | 3 | 3.5 | 0.03 | 2.14 | 70 |
| 53 | FLAIR | GE | SIGNA_Pioneer | WCMI_UPTOWN | 2017-06-23T15:13:51.000000 | 3 | 3 | 0.116816 | 9 | 125 |
| 53 | PD | GE | SIGNA_Pioneer | WCMI_UPTOWN | 2017-06-23T15:18:56.000000 | 3 | 3 | 0.00952 | 3.35 | 142 |
| 53 | T1w | GE | SIGNA_Pioneer | WCMI_UPTOWN | 2017-06-23T15:08:59.000000 | 3 | 1 | 0.003156 | 0.007364 | 12 |
| 53 | T2star | GE | SIGNA_Pioneer | WCMI_UPTOWN | 2017-06-23T15:23:46.000000 | 3 | 3 | 0.015 | 0.65 | 20 |
| 53 | T2w | GE | SIGNA_Pioneer | WCMI_UPTOWN | 2017-06-23T15:18:56.000000 | 3 | 3 | 0.08568 | 3.35 | 142 |
| 53 | DWI | GE | SIGNA_Pioneer | WCMI_UPTOWN | 2017-06-23T15:27:3.000000 | 3 | 2 | 0.0783 | 12.5 | 90 |
| 53 | rsfMRI | GE | SIGNA_Pioneer | WCMI_UPTOWN | 2017-06-23T15:34:48.000000 | 3 | 3.5 | 0.03 | 2.5 | 70 |
| 54 | FLAIR | GE | DISCOVERY_MR750 | Concordia_University_Perform_Ctr | 2017-09-01T09:41:32.000000 | 3 | 3 | 0.14378 | 9 | 125 |
| 54 | PD | GE | DISCOVERY_MR750 | Concordia_University_Perform_Ctr | 2017-09-01T09:37:28.000000 | 3 | 3 | 0.011224 | 3 | 125 |
| 54 | T1w | GE | DISCOVERY_MR750 | Concordia_University_Perform_Ctr | 2017-09-01T09:30:58.000000 | 3 | 1 | 0.002984 | 0.006884 | 11 |
| 54 | T2star | GE | DISCOVERY_MR750 | Concordia_University_Perform_Ctr | 2017-09-01T09:48:18.000000 | 3 | 3 | 0.02 | 0.65 | 20 |
| 54 | T2w | GE | DISCOVERY_MR750 | Concordia_University_Perform_Ctr | 2017-09-01T09:37:28.000000 | 3 | 3 | 0.089792 | 3 | 125 |
| 54 | DWI | GE | DISCOVERY_MR750 | Concordia_University_Perform_Ctr | 2017-09-01T09:55:10.000000 | 3 | 2 | 0.083 | 9 | 90 |
| 54 | rsfMRI | GE | DISCOVERY_MR750 | Concordia_University_Perform_Ctr | 2017-09-01T10:01:18.000000 | 3 | 3.5 | 0.03 | 2.4 | 70 |
| 55 | ToF | Philips | Achieva | IRM_Quebec_-_Mailloux_-_3T | 2017-12-20T12:50:36.380000 | 3 | 1 | 0.003454 | 0.025 | 20 |
| 55 | SWI | Philips | Achieva | IRM_Quebec_-_Mailloux_-_3T | 2017-12-20T12:45:35.010000 | 3 | 2 | 0.005403 | 0.0266077 | 15 |
| 55 | SWI | Philips | Achieva | IRM_Quebec_-_Mailloux_-_3T | 2017-12-20T12:45:35.010000 | 3 | 2 | 0.011322 | 0.0266077 | 15 |
| 55 | SWI | Philips | Achieva | IRM_Quebec_-_Mailloux_-_3T | 2017-12-20T12:45:35.010000 | 3 | 2 | 0.017241 | 0.0266077 | 15 |
| 55 | SWI | Philips | Achieva | IRM_Quebec_-_Mailloux_-_3T | 2017-12-20T12:45:35.010000 | 3 | 2 | 0.02316 | 0.0266077 | 15 |
| 55 | SWI | Philips | Achieva | IRM_Quebec_-_Mailloux_-_3T | 2017-12-20T12:45:35.010000 | 3 | 2 | 0.005403 | 0.0266077 | 15 |
| 55 | SWI | Philips | Achieva | IRM_Quebec_-_Mailloux_-_3T | 2017-12-20T12:45:35.010000 | 3 | 2 | 0.011322 | 0.0266077 | 15 |
| 55 | SWI | Philips | Achieva | IRM_Quebec_-_Mailloux_-_3T | 2017-12-20T12:45:35.010000 | 3 | 2 | 0.017241 | 0.0266077 | 15 |
| 55 | SWI | Philips | Achieva | IRM_Quebec_-_Mailloux_-_3T | 2017-12-20T12:45:35.010000 | 3 | 2 | 0.02316 | 0.0266077 | 15 |
| 55 | FLAIR | Philips | Achieva | IRM_Quebec_-_Mailloux_-_3T | 2017-12-20T12:57:2.310000 | 3 | 3 | 0.125 | 9 | 90 |
| 55 | PD | Philips | Achieva | IRM_Quebec_-_Mailloux_-_3T | 2017-12-20T12:23:20.850000 | 3 | 3 | 0.0125 | 3 | 90 |
| 55 | T1w | Philips | Achieva | IRM_Quebec_-_Mailloux_-_3T | 2017-12-20T12:16:32.190000 | 3 | 1 | 0.003313 | 0.0073029 | 9 |
| 55 | T2star | Philips | Achieva | IRM_Quebec_-_Mailloux_-_3T | 2017-12-20T13:00:39.290000 | 3 | 3 | 0.020722 | 0.65 | 20 |
| 55 | T2w | Philips | Achieva | IRM_Quebec_-_Mailloux_-_3T | 2017-12-20T12:23:20.850000 | 3 | 3 | 0.1 | 3 | 90 |
| 55 | DWI | Philips | Achieva | IRM_Quebec_-_Mailloux_-_3T | 2017-12-20T12:29:7.370000 | 3 | 2 | 0.097086 | 9.51422 | 90 |
| 55 | rsfMRI | Philips | Achieva | IRM_Quebec_-_Mailloux_-_3T | 2017-12-20T12:36:30.320000 | 3 | 3.5 | 0.030001 | 2.11 | 70 |
| 56 | FLAIR | Philips | Ingenia | CHUM | 2018-03-13T10:50:38.980000 | 3 | 3 | 0.125 | 9 | 90 |
| 56 | PD | Philips | Ingenia | CHUM | 2018-03-13T10:44:50.170000 | 3 | 3 | 0.0125 | 3 | 90 |
| 56 | T1w | Philips | Ingenia | CHUM | 2018-03-13T10:38:23.560000 | 3 | 1 | 0.003347 | 0.0073415 | 9 |
| 56 | T2star | Philips | Ingenia | CHUM | 2018-03-13T10:54:18.070000 | 3 | 3 | 0.020719 | 0.65 | 20 |
| 56 | T2w | Philips | Ingenia | CHUM | 2018-03-13T10:44:50.170000 | 3 | 3 | 0.1 | 3 | 90 |
| 56 | DWI | Philips | Ingenia | CHUM | 2018-03-13T11:08:36.510000 | 3 | 2 | 0.109 | 10.2665 | 90 |
| 56 | rsfMRI | Philips | Ingenia | CHUM | 2018-03-13T10:59:9.960000 | 3 | 3.5 | 0.030001 | 2.11 | 70 |
| 57 | FLAIR | Siemens | Prisma_fit | The_Hospital_for_Sick_Children | 2018-06-04T08:59:30.475000 | 3 | 3 | 0.12 | 9 | 165 |
| 57 | PD | Siemens | Prisma_fit | The_Hospital_for_Sick_Children | 2018-06-04T08:56:10.992500 | 3 | 3 | 0.01 | 3 | 165 |
| 57 | T1w | Siemens | Prisma_fit | The_Hospital_for_Sick_Children | 2018-06-04T08:50:14.967500 | 3 | 1 | 0.00298 | 2.3 | 9 |
| 57 | T2star | Siemens | Prisma_fit | The_Hospital_for_Sick_Children | 2018-06-04T09:03:36.990000 | 3 | 3 | 0.0044 | 0.65 | 20 |
| 57 | T2w | Siemens | Prisma_fit | The_Hospital_for_Sick_Children | 2018-06-04T08:56:11.085000 | 3 | 3 | 0.093 | 3 | 165 |
| 57 | DWI | Siemens | Prisma_fit | The_Hospital_for_Sick_Children | 2018-06-04T09:16:44.367500 | 3 | 2 | 0.064 | 9.4 | 90 |
| 57 | DWI | Siemens | Prisma_fit | The_Hospital_for_Sick_Children | 2018-06-04T09:24:54.137500 | 3 | 2 | 0.064 | 9.4 | 90 |
| 57 | rsfMRI | Siemens | Prisma_fit | The_Hospital_for_Sick_Children | 2018-06-04T09:05:54.280000 | 3 | 3.5 | 0.03 | 2.4 | 70 |
| 58 | T2star | Siemens | Prisma_fit | York_MRI_Facility | 2018-06-04T11:48:1.330000 | 3 | 3 | 0.02 | 0.65 | 20 |
| 58 | FLAIR | Siemens | Prisma_fit | York_MRI_Facility | 2018-06-04T11:44:22.130000 | 3 | 3 | 0.12 | 9 | 165 |
| 58 | PD | Siemens | Prisma_fit | York_MRI_Facility | 2018-06-04T11:40:52.510000 | 3 | 3 | 0.011 | 3 | 165 |
| 58 | T1w | Siemens | Prisma_fit | York_MRI_Facility | 2018-06-04T11:34:56.195000 | 3 | 1 | 0.00298 | 2.3 | 9 |
| 58 | T2star | Siemens | Prisma_fit | York_MRI_Facility | 2018-06-04T11:48:1.330000 | 3 | 3 | 0.02 | 0.65 | 20 |
| 58 | T2w | Siemens | Prisma_fit | York_MRI_Facility | 2018-06-04T11:40:52.587500 | 3 | 3 | 0.089 | 3 | 165 |
| 58 | DWI | Siemens | Prisma_fit | York_MRI_Facility | 2018-06-04T12:01:2.635000 | 3 | 2 | 0.096 | 9.4 | 90 |
| 58 | rsfMRI | Siemens | Prisma_fit | York_MRI_Facility | 2018-06-04T11:50:12.527500 | 3 | 3.5 | 0.03 | 2.4 | 70 |
| 59 | FLAIR | Siemens | Skyra | St._Michael`s_Hospital | 2018-06-05T08:50:23.477500 | 3 | 3 | 0.12 | 9 | 165 |
| 59 | PD | Siemens | Skyra | St._Michael`s_Hospital | 2018-06-05T08:46:37.965000 | 3 | 3 | 0.011 | 3 | 165 |
| 59 | T1w | Siemens | Skyra | St._Michael`s_Hospital | 2018-06-05T08:40:45.320000 | 3 | 1 | 0.00298 | 2.3 | 9 |
| 59 | T2star | Siemens | Skyra | St._Michael`s_Hospital | 2018-06-05T08:54:10.187500 | 3 | 3 | 0.02 | 0.65 | 20 |
| 59 | T2w | Siemens | Skyra | St._Michael`s_Hospital | 2018-06-05T08:46:38.060000 | 3 | 3 | 0.095 | 3 | 165 |
| 59 | DWI | Siemens | Skyra | St._Michael`s_Hospital | 2018-06-05T09:06:54.927500 | 3 | 2 | 0.096 | 9.5 | 90 |
| 59 | rsfMRI | Siemens | Skyra | St._Michael`s_Hospital | 2018-06-05T08:56:1.182500 | 3 | 3.5 | 0.03 | 2.4 | 70 |
| 60 | T2star | GE | DISCOVERY_MR750 | CAMH | 2018-06-05T11:50:45.000000 | 3 | 3 | 0.02 | 0.65 | 20 |
| 60 | FLAIR | GE | DISCOVERY_MR750 | CAMH | 2018-06-05T11:42:3.000000 | 3 | 3 | 0.140476 | 9 | 125 |
| 60 | PD | GE | DISCOVERY_MR750 | CAMH | 2018-06-05T11:38:39.000000 | 3 | 3 | 0.010712 | 3 | 125 |
| 60 | T1w | GE | DISCOVERY_MR750 | CAMH | 2018-06-05T11:34:7.000000 | 3 | 1 | 0.00294 | 0.00668 | 11 |
| 60 | T2star | GE | DISCOVERY_MR750 | CAMH | 2018-06-05T11:48:8.000000 | 3 | 3 | 0.02 | 0.65 | 20 |
| 60 | T2w | GE | DISCOVERY_MR750 | CAMH | 2018-06-05T11:38:39.000000 | 3 | 3 | 0.085696 | 3 | 125 |
| 60 | rsfMRI | GE | DISCOVERY_MR750 | CAMH | 2018-06-05T11:56:33.000000 | 3 | 3.5 | 0.03 | 2.4 | 70 |
| 61 | FLAIR | Siemens | TrioTim | Hospital_Douglas | 2018-06-11T10:04:5.990000 | 3 | 3 | 0.123 | 9 | 165 |
| 61 | PD | Siemens | TrioTim | Hospital_Douglas | 2018-06-11T10:08:16.687500 | 3 | 3 | 0.01 | 3 | 165 |
| 61 | T1w | Siemens | TrioTim | Hospital_Douglas | 2018-06-11T09:58:12.212500 | 3 | 1 | 0.00298 | 2.3 | 9 |
| 61 | T2star | Siemens | TrioTim | Hospital_Douglas | 2018-06-11T10:15:8.635000 | 3 | 3 | 0.02 | 0.65 | 20 |
| 61 | T2w | Siemens | TrioTim | Hospital_Douglas | 2018-06-11T10:08:16.780000 | 3 | 3 | 0.091 | 3 | 165 |
| 61 | FLAIR | Siemens | TrioTim | Hospital_Douglas | 2018-06-11T10:39:14.035000 | 3 | 3 | 0.123 | 9 | 165 |
| 61 | PD | Siemens | TrioTim | Hospital_Douglas | 2018-06-11T10:43:14.097500 | 3 | 3 | 0.01 | 3 | 165 |
| 61 | T1w | Siemens | TrioTim | Hospital_Douglas | 2018-06-11T10:33:30.582500 | 3 | 1 | 0.00298 | 2.3 | 9 |
| 61 | T2star | Siemens | TrioTim | Hospital_Douglas | 2018-06-11T10:50:5.520000 | 3 | 3 | 0.02 | 0.65 | 20 |
| 61 | T2w | Siemens | TrioTim | Hospital_Douglas | 2018-06-11T10:43:14.190000 | 3 | 3 | 0.091 | 3 | 165 |
| 61 | DWI | Siemens | TrioTim | Hospital_Douglas | 2018-06-11T10:17:23.805000 | 3 | 2 | 0.096 | 9.4 | 90 |
| 61 | DWI | Siemens | TrioTim | Hospital_Douglas | 2018-06-11T10:52:21.355000 | 3 | 2 | 0.096 | 9.4 | 90 |
| 61 | rsfMRI | Siemens | TrioTim | Hospital_Douglas | 2018-06-11T10:24:3.860000 | 3 | 3.5 | 0.03 | 2.11 | 70 |
| 61 | rsfMRI | Siemens | TrioTim | Hospital_Douglas | 2018-06-11T10:59:0.702500 | 3 | 3.5 | 0.03 | 2.11 | 70 |
| 62 | FLAIR | Siemens | Prisma_fit | Mc_Connell_Brain_Imaging_Centre | 2018-06-11T12:17:15.465000 | 3 | 3 | 0.12 | 9 | 165 |
| 62 | PD | Siemens | Prisma_fit | Mc_Connell_Brain_Imaging_Centre | 2018-06-11T12:13:53.977500 | 3 | 3 | 0.01 | 3 | 165 |
| 62 | T1w | Siemens | Prisma_fit | Mc_Connell_Brain_Imaging_Centre | 2018-06-11T12:07:21.462500 | 3 | 1 | 0.00298 | 2.3 | 9 |
| 62 | T2star | Siemens | Prisma_fit | Mc_Connell_Brain_Imaging_Centre | 2018-06-11T12:21:21.002500 | 3 | 3 | 0.02 | 0.65 | 20 |
| 62 | T2w | Siemens | Prisma_fit | Mc_Connell_Brain_Imaging_Centre | 2018-06-11T12:13:54.070000 | 3 | 3 | 0.093 | 3 | 165 |
| 62 | DWI | Siemens | Prisma_fit | Mc_Connell_Brain_Imaging_Centre | 2018-06-11T12:23:43.245000 | 3 | 2 | 0.064 | 6.9 | 90 |
| 62 | rsfMRI | Siemens | Prisma_fit | Mc_Connell_Brain_Imaging_Centre | 2018-06-11T12:28:6.475000 | 3 | 3.5 | 0.03 | 2.13 | 70 |
| 63 | FLAIR | Siemens | Prisma_fit | IUGM | 2018-06-11T15:44:18.760000 | 3 | 3 | 0.12 | 9 | 165 |
| 63 | PD | Siemens | Prisma_fit | IUGM | 2018-06-11T15:40:55.212500 | 3 | 3 | 0.01 | 3 | 165 |
| 63 | T1w | Siemens | Prisma_fit | IUGM | 2018-06-11T15:34:28.785000 | 3 | 1 | 0.00298 | 2.3 | 9 |
| 63 | T2star | Siemens | Prisma_fit | IUGM | 2018-06-11T15:48:29.480000 | 3 | 3 | 0.02 | 0.65 | 20 |
| 63 | T2w | Siemens | Prisma_fit | IUGM | 2018-06-11T15:40:55.305000 | 3 | 3 | 0.093 | 3 | 165 |
| 63 | DWI | Siemens | Prisma_fit | IUGM | 2018-06-11T15:50:42.167500 | 3 | 2 | 0.064 | 6.9 | 90 |
| 63 | rsfMRI | Siemens | Prisma_fit | IUGM | 2018-06-11T15:56:1.162500 | 3 | 3.5 | 0.03 | 2.13 | 70 |
| 64 | FLAIR | Siemens | TrioTim | THE_OTTAWA_HOSPITAL_CIVIC | 2018-06-13T14:26:20.305000 | 3 | 3 | 0.123 | 9 | 165 |
| 64 | PD | Siemens | TrioTim | THE_OTTAWA_HOSPITAL_CIVIC | 2018-06-13T14:30:32.022500 | 3 | 3 | 0.01 | 3 | 165 |
| 64 | T1w | Siemens | TrioTim | THE_OTTAWA_HOSPITAL_CIVIC | 2018-06-13T14:20:22.515000 | 3 | 1 | 0.00298 | 2.3 | 9 |
| 64 | T2star | Siemens | TrioTim | THE_OTTAWA_HOSPITAL_CIVIC | 2018-06-13T14:37:20.710000 | 3 | 3 | 0.02 | 0.65 | 20 |
| 64 | T2w | Siemens | TrioTim | THE_OTTAWA_HOSPITAL_CIVIC | 2018-06-13T14:30:32.112500 | 3 | 3 | 0.091 | 3 | 165 |
| 64 | DWI | Siemens | TrioTim | THE_OTTAWA_HOSPITAL_CIVIC | 2018-06-13T14:39:33.097500 | 3 | 2 | 0.096 | 9.4 | 90 |
| 64 | rsfMRI | Siemens | TrioTim | THE_OTTAWA_HOSPITAL_CIVIC | 2018-06-13T14:46:23.292500 | 3 | 3.5 | 0.03 | 2.11 | 70 |
| 65 | FLAIR | Siemens | TrioTim | Queens_University | 2018-06-24T13:25:43.257500 | 3 | 3 | 0.119 | 9 | 165 |
| 65 | PD | Siemens | TrioTim | Queens_University | 2018-06-24T13:22:16.865000 | 3 | 3 | 0.011 | 3 | 165 |
| 65 | T1w | Siemens | TrioTim | Queens_University | 2018-06-24T13:15:46.202500 | 3 | 1 | 0.00197 | 2.3 | 9 |
| 65 | T2star | Siemens | TrioTim | Queens_University | 2018-06-24T13:28:57.060000 | 3 | 3 | 0.02 | 0.65 | 20 |
| 65 | T2w | Siemens | TrioTim | Queens_University | 2018-06-24T13:22:16.942500 | 3 | 3 | 0.088 | 3 | 165 |
| 65 | DWI | Siemens | TrioTim | Queens_University | 2018-06-24T13:41:41.707500 | 3 | 2 | 0.096 | 9.4 | 90 |
| 65 | rsfMRI | Siemens | TrioTim | Queens_University | 2018-06-24T13:30:40.497500 | 3 | 3.5 | 0.03 | 2.4 | 70 |
| 66 | FLAIR | GE | DISCOVERY_MR750 | ST_JOSEPH_HAMILTON | 2018-06-27T15:35:20.000000 | 3 | 3 | 0.140476 | 9 | 125 |
| 66 | PD | GE | DISCOVERY_MR750 | ST_JOSEPH_HAMILTON | 2018-06-27T15:27:56.000000 | 3 | 3 | 0.010712 | 3 | 125 |
| 66 | T1w | GE | DISCOVERY_MR750 | ST_JOSEPH_HAMILTON | 2018-06-27T15:22:1.000000 | 3 | 1 | 0.00318 | 0.008156 | 11 |
| 66 | T2star | GE | DISCOVERY_MR750 | ST_JOSEPH_HAMILTON | 2018-06-27T15:40:44.000000 | 3 | 3 | 0.02 | 0.65 | 20 |
| 66 | T2w | GE | DISCOVERY_MR750 | ST_JOSEPH_HAMILTON | 2018-06-27T15:27:56.000000 | 3 | 3 | 0.085696 | 3 | 125 |
| 66 | PD | GE | DISCOVERY_MR750 | ST_JOSEPH_HAMILTON | 2018-06-27T15:31:52.000000 | 3 | 3 | 0.010712 | 3 | 125 |
| 66 | T2w | GE | DISCOVERY_MR750 | ST_JOSEPH_HAMILTON | 2018-06-27T15:31:52.000000 | 3 | 3 | 0.085696 | 3 | 125 |
| 66 | DWI | GE | DISCOVERY_MR750 | ST_JOSEPH_HAMILTON | 2018-06-27T15:43:22.000000 | 3 | 2 | 0.0821 | 9 | 90 |
| 66 | rsfMRI | GE | DISCOVERY_MR750 | ST_JOSEPH_HAMILTON | 2018-06-27T15:50:20.000000 | 3 | 3.5 | 0.03 | 2.4 | 70 |
| 67 | T2star | Siemens | Prisma_fit | Robarts_Research_Institute | 2018-06-29T09:07:9.975000 | 3 | 3 | 0.02 | 0.65 | 20 |
| 67 | FLAIR | Siemens | Prisma_fit | Robarts_Research_Institute | 2018-06-29T09:03:4.462500 | 3 | 3 | 0.12 | 9 | 165 |
| 67 | PD | Siemens | Prisma_fit | Robarts_Research_Institute | 2018-06-29T08:59:42.700000 | 3 | 3 | 0.01 | 3 | 165 |
| 67 | T1w | Siemens | Prisma_fit | Robarts_Research_Institute | 2018-06-29T08:54:5.445000 | 3 | 1 | 0.00298 | 2.3 | 9 |
| 67 | T2star | Siemens | Prisma_fit | Robarts_Research_Institute | 2018-06-29T09:07:9.975000 | 3 | 3 | 0.02 | 0.65 | 20 |
| 67 | T2w | Siemens | Prisma_fit | Robarts_Research_Institute | 2018-06-29T08:59:42.792500 | 3 | 3 | 0.093 | 3 | 165 |
| 67 | DWI | Siemens | Prisma_fit | Robarts_Research_Institute | 2018-06-29T09:18:49.235000 | 3 | 2 | 0.064 | 6.9 | 90 |
| 67 | rsfMRI | Siemens | Prisma_fit | Robarts_Research_Institute | 2018-06-29T09:09:19.247500 | 3 | 3.5 | 0.03 | 2.13 | 70 |
| 68 | FLAIR | Siemens | Skyra | 3T_ROYAL_UNIVERSITY_HOSP | 2018-07-03T17:50:21.840000 | 3 | 3 | 0.125 | 9 | 165 |
| 68 | PD | Siemens | Skyra | 3T_ROYAL_UNIVERSITY_HOSP | 2018-07-03T17:54:27.405000 | 3 | 3 | 0.01 | 3 | 165 |
| 68 | T1w | Siemens | Skyra | 3T_ROYAL_UNIVERSITY_HOSP | 2018-07-03T17:43:34.435000 | 3 | 1 | 0.00298 | 2.3 | 9 |
| 68 | T2star | Siemens | Skyra | 3T_ROYAL_UNIVERSITY_HOSP | 2018-07-03T18:01:29.425000 | 3 | 3 | 0.02 | 0.65 | 20 |
| 68 | T2w | Siemens | Skyra | 3T_ROYAL_UNIVERSITY_HOSP | 2018-07-03T17:54:27.497500 | 3 | 3 | 0.093 | 3 | 165 |
| 68 | DWI | Siemens | Skyra | 3T_ROYAL_UNIVERSITY_HOSP | 2018-07-03T18:17:57.312500 | 3 | 2 | 0.101 | 13 | 90 |
| 68 | rsfMRI | Siemens | Skyra | 3T_ROYAL_UNIVERSITY_HOSP | 2018-07-03T18:03:17.722500 | 3 | 3.5 | 0.03 | 2.14 | 70 |
| 68 | rsfMRI | Siemens | Skyra | 3T_ROYAL_UNIVERSITY_HOSP | 2018-07-03T18:07:52.197500 | 3 | 3.5 | 0.03 | 2.14 | 70 |
| 69 | T2star | Siemens | Prisma | Peter_S._Allen_MR_Research_Centre | 2018-07-04T15:33:5.727500 | 3 | 3 | 0.02 | 0.65 | 20 |
| 69 | FLAIR | Siemens | Prisma | Peter_S._Allen_MR_Research_Centre | 2018-07-04T15:28:55.442500 | 3 | 3 | 0.12 | 9 | 165 |
| 69 | PD | Siemens | Prisma | Peter_S._Allen_MR_Research_Centre | 2018-07-04T15:25:30.802500 | 3 | 3 | 0.01 | 3 | 165 |
| 69 | T1w | Siemens | Prisma | Peter_S._Allen_MR_Research_Centre | 2018-07-04T15:18:35.605000 | 3 | 1 | 0.00298 | 2.3 | 9 |
| 69 | T2star | Siemens | Prisma | Peter_S._Allen_MR_Research_Centre | 2018-07-04T15:33:5.727500 | 3 | 3 | 0.02 | 0.65 | 20 |
| 69 | T2w | Siemens | Prisma | Peter_S._Allen_MR_Research_Centre | 2018-07-04T15:25:30.895000 | 3 | 3 | 0.093 | 3 | 165 |
| 69 | DWI | Siemens | Prisma | Peter_S._Allen_MR_Research_Centre | 2018-07-04T15:35:14.845000 | 3 | 2 | 0.064 | 6.9 | 90 |
| 69 | rsfMRI | Siemens | Prisma | Peter_S._Allen_MR_Research_Centre | 2018-07-04T15:39:37.227500 | 3 | 3.5 | 0.03 | 2.13 | 70 |
| 70 | ASL | GE | DISCOVERY_MR750 | FOOTHILLS_Hospital | 2018-07-05T16:46:43.000000 | 3 | 3.5 | 0.010804 | 5.382 | 111 |
| 70 | FLAIR | GE | DISCOVERY_MR750 | FOOTHILLS_Hospital | 2018-07-05T16:22:46.000000 | 3 | 3 | 0.14378 | 9 | 125 |
| 70 | PD | GE | DISCOVERY_MR750 | FOOTHILLS_Hospital | 2018-07-05T16:19:40.000000 | 3 | 3 | 0.011224 | 3 | 125 |
| 70 | T1w | GE | DISCOVERY_MR750 | FOOTHILLS_Hospital | 2018-07-05T16:12:14.000000 | 3 | 1 | 0.003052 | 0.00738 | 11 |
| 70 | T2star | GE | DISCOVERY_MR750 | FOOTHILLS_Hospital | 2018-07-05T16:28:16.000000 | 3 | 3 | 0.02 | 0.65 | 20 |
| 70 | T2w | GE | DISCOVERY_MR750 | FOOTHILLS_Hospital | 2018-07-05T16:19:40.000000 | 3 | 3 | 0.089792 | 3 | 125 |
| 70 | DWI | GE | DISCOVERY_MR750 | FOOTHILLS_Hospital | 2018-07-05T16:31:43.000000 | 3 | 2 | 0.0833 | 9 | 90 |
| 70 | rsfMRI | GE | DISCOVERY_MR750 | FOOTHILLS_Hospital | 2018-07-05T16:37:33.000000 | 3 | 3.5 | 0.03 | 2.5 | 70 |
| 71 | FLAIR | Philips | Intera | UBC_MRI_Research_Centre | 2018-07-09T15:31:31.960000 | 3 | 3 | 0.125 | 9 | 90 |
| 71 | PD | Philips | Intera | UBC_MRI_Research_Centre | 2018-07-09T15:25:31.870000 | 3 | 3 | 0.0125 | 3 | 90 |
| 71 | T1w | Philips | Intera | UBC_MRI_Research_Centre | 2018-07-09T15:17:24.510000 | 3 | 1 | 0.00327 | 0.0072162 | 9 |
| 71 | T2star | Philips | Intera | UBC_MRI_Research_Centre | 2018-07-09T15:36:35.890000 | 3 | 3 | 0.020713 | 0.65 | 20 |
| 71 | T2w | Philips | Intera | UBC_MRI_Research_Centre | 2018-07-09T15:25:31.870000 | 3 | 3 | 0.1 | 3 | 90 |
| 71 | DWI | Philips | Intera | UBC_MRI_Research_Centre | 2018-07-09T15:42:9.790000 | 3 | 2 | 0.100347 | 9.93249 | 90 |
| 71 | rsfMRI | Philips | Intera | UBC_MRI_Research_Centre | 2018-07-09T15:48:56.200000 | 3 | 3.5 | 0.03 | 2.11 | 70 |
| 72 | FLAIR | GE | SIGNA_Pioneer | WCMI_UPTOWN | 2018-07-12T14:25:46.000000 | 3 | 3 | 0.117488 | 9 | 125 |
| 72 | PD | GE | SIGNA_Pioneer | WCMI_UPTOWN | 2018-07-12T14:31:53.000000 | 3 | 3 | 0.009528 | 3.35 | 142 |
| 72 | T1w | GE | SIGNA_Pioneer | WCMI_UPTOWN | 2018-07-12T14:19:55.000000 | 3 | 1 | 0.003156 | 0.007364 | 12 |
| 72 | T2star | GE | SIGNA_Pioneer | WCMI_UPTOWN | 2018-07-12T14:42:24.000000 | 3 | 3 | 0.015 | 0.65 | 20 |
| 72 | T2w | GE | SIGNA_Pioneer | WCMI_UPTOWN | 2018-07-12T14:31:53.000000 | 3 | 3 | 0.085752 | 3.35 | 142 |
| 72 | DWI | GE | SIGNA_Pioneer | WCMI_UPTOWN | 2018-07-12T14:46:39.000000 | 3 | 3 | 0.0819 | 12.5 | 90 |
| 72 | rsfMRI | GE | SIGNA_Pioneer | WCMI_UPTOWN | 2018-07-12T14:54:24.000000 | 3 | 3.5 | 0.03 | 2.5 | 70 |
| 73 | FLAIR | Philips | Achieva_dStream | IRM_Quebec_-_Mailloux_-_3T | 2018-09-18T13:49:26.040000 | 3 | 3 | 0.125 | 9 | 90 |
| 73 | PD | Philips | Achieva_dStream | IRM_Quebec_-_Mailloux_-_3T | 2018-09-18T13:44:0.520000 | 3 | 3 | 0.0125 | 3 | 90 |
| 73 | T1w | Philips | Achieva_dStream | IRM_Quebec_-_Mailloux_-_3T | 2018-09-18T13:37:17.040000 | 3 | 1 | 0.003299 | 0.0072814 | 9 |
| 73 | T2star | Philips | Achieva_dStream | IRM_Quebec_-_Mailloux_-_3T | 2018-09-18T13:52:59.560000 | 3 | 3 | 0.020719 | 0.65 | 20 |
| 73 | T2w | Philips | Achieva_dStream | IRM_Quebec_-_Mailloux_-_3T | 2018-09-18T13:44:0.520000 | 3 | 3 | 0.1 | 3 | 90 |
| 73 | DWI | Philips | Achieva_dStream | IRM_Quebec_-_Mailloux_-_3T | 2018-09-18T13:57:44.980000 | 3 | 2 | 0.097299 | 9.52664 | 90 |
| 73 | rsfMRI | Philips | Achieva_dStream | IRM_Quebec_-_Mailloux_-_3T | 2018-09-18T14:04:40.100000 | 3 | 3.5 | 0.030001 | 2.11 | 70 |
| ASL: Arterial spin labeling. DWI: Diffusion-weighted imaging. PD: Proton density imaging. SWI: Susceptibility-weighted imaging. TE: Echo time. ToF: Time of Flight. TR: Repetition time. rsfMRI: Resting state functional MRI. | | | | | | | | | | |
